# qSOFA is a Poor Predictor of Short-Term Mortality in All Patients: A Systematic Review of 410,000 Patients

**DOI:** 10.3390/jcm8010061

**Published:** 2019-01-08

**Authors:** Ronson S. L. Lo, Ling Yan Leung, Mikkel Brabrand, Chun Yu Yeung, Suet Yi Chan, Cherry C. Y. Lam, Kevin K. C. Hung, Colin A. Graham

**Affiliations:** 1 Accident and Emergency Medicine Academic Unit, Chinese University of Hong Kong, Hong Kong, China; ronsonsllo@cuhk.edu.hk (R.S.L.L.); lingleung@cuhk.edu.hk (L.Y.L.); Mikkel.Brabrand@rsyd.dk (M.B.); gregory_ycyg@yahoo.com.hk (C.Y.Y.); chan_syi@hotmail.com (S.Y.C.); 1155108119@link.cuhk.edu.hk (C.C.Y.L.); kevin.hung@cuhk.edu.hk (K.K.C.H.); 2 Department of Emergency Medicine, Hospital of South West Denmark, Finsensgade 35, DK-6700 Esbjerg, Denmark

**Keywords:** sepsis, qSOFA, prognosis

## Abstract

Background: To determine the validity of the Quick Sepsis-Related Organ Failure Assessment (qSOFA) in the prediction of outcome (in-hospital and 1-month mortality, intensive care unit (ICU) admission, and hospital and ICU length of stay) in adult patients with or without suspected infections where qSOFA was calculated and reported; Methods: Cochrane Central of Controlled trials, EMBASE, BIOSIS, OVID MEDLINE, OVID Nursing Database, and the Joanna Briggs Institute EBP Database were the main databases searched. All studies published until 12 April 2018 were considered. All studies except case series, case reports, and conference abstracts were considered. Studies that included patients with neutropenic fever exclusively were excluded. Results: The median AUROC for in-hospital mortality (27 studies with 380,920 patients) was 0.68 (a range of 0.55 to 0.82). A meta-analysis of 377,623 subjects showed a polled AUROC of 0.68 (0.65 to 0.71); however, it also confirmed high heterogeneity among studies (I^2^ = 98.8%, 95%CI 98.6 to 99.0). The median sensitivity and specificity for in-hospital mortality (24 studies with 118,051 patients) was 0.52 (range 0.16 to 0.98) and 0.81 (0.19 to 0.97), respectively. Median positive and negative predictive values were 0.2 (range 0.07 to 0.38) and 0.94 (0.85 to 0.99), respectively.

## 1. Introduction

Sepsis has been the focus of intensive research efforts over many years, with good reason [1]. Mortality is high (as high as 28.6% [2]) and treatment is expensive ($18,600 USD per hospital stay in the US [3]).

The first international consensus definition of sepsis dates from 1992 [4,5]. It was not substantially updated until 2016 [6] when the task group for the third international consensus definition for sepsis and septic shock redefined sepsis as a “life-threatening organ dysfunction caused by a dysregulated host response to infection” [6] Alongside with this updated definition, the task group also proposed a novel score to identify patients at risk for sepsis: the Quick Sepsis-Related Organ Failure Assessment (qSOFA). However, like many changes, qSOFA has been controversial [7,8,9].

qSOFA was based on the Sepsis-related Organ Failure Assessment (SOFA) score. The SOFA score was originally developed as a predictor for intensive care unit (ICU) mortality [10], and it consists of both vital signs (respiratory rate and blood pressure) and laboratory assessments (liver function tests, urea and creatinine) [6]. qSOFA was intended for use in patients with suspected infection outside of the ICU setting, and included altered mentation, tachypnea, and hypotension [6].

Prior systematic reviews on the topic tend to focus on patients that have already been identified as having suspected infections, which is how the test was originally designed. However, in an Emergency department (ED), the cause for attendance is not always clear, and a diagnosis of infection is often made much later. We there believe that qSOFA should be applied earlier in the treatment process, before a specific condition is considered. This systematic review aims to determine the validity of qSOFA in the prediction of mortality in all patients, with or without a suspected infection.

Objectives: This systematic review examines the validity of qSOFA in predicting in-hospital mortality and 28/30-days mortality, and determines if qSOFA is able to predict ICU admission, length of ICU stay, length of hospital stay, and diagnosis of sepsis, in patients not already identified with a specific condition.

## 2. Methodology

We designed our systematic review using the framework set out in the Preferred Reporting Items for Systematic Reviews and Meta-Analysis Protocols (PRISMA-P) 2015 statement developed with elements adapted from the Cochrane Handbook for Systematic Reviews of Interventions [11,12]. The review was registered with PROSPERO (ID CRD42017063976).

### 2.1. Eligibility Criteria

Types of studies: We considered studies of all designs, except for case series and case reports, i.e., all retrospective and prospective, and all observational and interventional studies. Studies only reported as abstracts were excluded.

Types of participants: All studies with adult patients with or without suspected or confirmed infection, sepsis, severe sepsis, and septic shock were considered. Studies that only included patients with neutropenic fever were excluded from this systematic review, due to the specific nature of this patient group.

Interventions: We considered all studies that reported qSOFA.

Setting: We found studies including patients presenting acutely to Emergency departments and pre-hospital emergency care providers, critical care units (intensive care units and high dependency units), and general wards.

Types of outcome: In-hospital mortality, 1-month mortality, ICU admission, diagnosis of sepsis, length of ICU stay, and length of hospital stay.

Timing: Both retrospective and prospective studies were considered.

Period of review: All studies published until 12 April 2018 were included.

Language: We included articles in languages that the author group could understand (English, Chinese, Danish). Papers with titles that seemed relevant but in languages that were non-comprehensible to the authors are listed in Appendix A (non-English studies).

### 2.2. Information Sources

Our literature search strategy was developed by using Medical Subject Headings (MeSH) and text words related to qSOFA. We searched the Cochrane Central Register of Controlled Trials (November 2016), EMBASE (1910 to Present), BIOSIS (2001 to 2012), OVID MEDLINE^®^ Epub Ahead of Print, In-Process & Other Non-Indexed Citations and OVID MEDLINE^®^ (1946 to Present with Daily Update), OVID Nursing Database (1946 to January Week 1 2017), and the Joanna Briggs Institute EBP Database, using the OVID interface. The WHO International Clinical Trial Registry Platform, Web of Science, Scopus, and ClinicalTrials.gov were searched independently.

### 2.3. Search Strategy

We have used the following terms to search ((((qSOFA) OR quick SOFA) OR quick sequential organ failure assessment) OR quick sepsis-related organ failure assessment) AND mortality.

Details may be found in Appendix B, Appendix C, Appendix D, Appendix E and Appendix F (search strategies).

### 2.4. Study Selection

Duplicates were removed, and records were identified and screened by LL and RL. After this, studies with no results available and studies in languages that our group could not read were also excluded. The remaining studies were discussed in a consensus meeting by CAG, MB, KH, LL, and RL. The results were compared at each stage, and discrepancies were discussed. If no consensus was met, CAG acted as the final adjudicator for the decision of whether a study should be included.

### 2.5. Data

Data was collected independently and was cross-checked by at least three reviewers. The data items extracted included study type (retrospective/prospective), sample size, patient characteristics such as age and gender, recruitment period, patient setting (location of recruitment), patient group (infection/‘all-comers’), mentation assessment, and the timing of qSOFA.

### 2.6. Outcomes

Our primary outcome was in-hospital mortality. Secondary outcomes were 1-month mortality, ICU admission, sepsis diagnosis, ICU length-of-stay, and hospital length-of-stay. We performed sub-group analyses for studies that only included patients with infection versus all-comers, the location of recruitment, altered mental status, and timing of qSOFA.

Graphs were generated using MedCalc Statistical Software version 18.11 [13].

### 2.7. Risk of Bias in Individual Studies

All studies included were assessed by using an adapted version of the Quality in Prognosis Studies instrument [14]. Six potential bias domains were explored: selection bias, bias in definition and measurement, outcome measurement bias, handling of missing data, confounding, and bias of statistics or the presentation of result. These six domains were be graded as “high risk (of bias)”, “low risk (of bias)”, or “unclear”.

Summary measures: The principal summary measure was the area under the receiver operator characteristic (AUROC) curve for the prediction of mortality. Sensitivity, specificity, positive predictive value (PPV), and negative predictive value (NPV) were also collected. All measures were also reported for Intensive Care Units (ICU) admission and sepsis diagnosis.

## 3. Results

### 3.1. Study Selection

**Figure 1:**
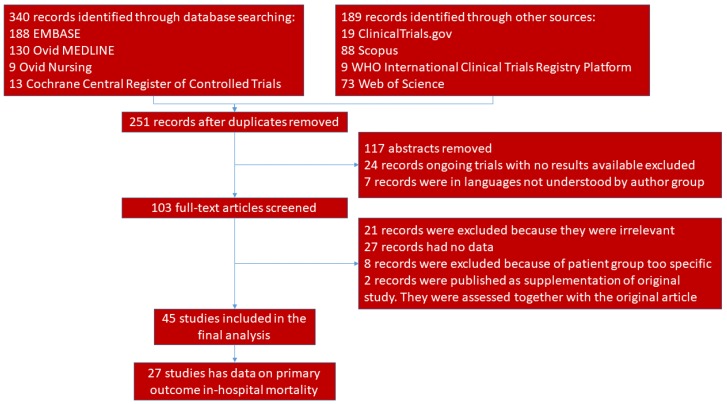
Study Flow.

The database search identified 529 records. After duplicates were removed, 251 records were identified and screened by LL and RL. After 117 abstracts were excluded, 24 ongoing trials with no results available, and seven records in languages that our group could not read were also excluded (all seven of these papers appeared to be reviews or articles that contained no original data). The remaining 103 were discussed in a consensus meeting by CAG, MB, KH, LL, and RL. We included 45 papers in the final analysis [15,16,17,18,19,20,21,22,23,24,25,26,27,28,29,30,31,32,33,34,35,36,37,38,39,40,41,42,43,44,45,46,47,48,49,50,51,52,53,54,55,56,57,58,59] (Figure 1). Excluded studies and the reasons for their exclusion are listed in Appendix G (Table A1).

### 3.2. Study and Sample Characteristics

Of the 45 studies, 27 were retrospective cohorts, 13 had data prospectively collected but retrospectively analyzed, and five were prospective cohorts. The studies recruited a total of 413,634 patients from Europe, North America, Asia, and Australasia, with a median age ranging from 49 to 80 years. Seven studies recruited patients from all settings, 24 studies recruited only ED patients, eight from ICU only, one from all non-ICU settings, one from general wards, one from a pre-hospital setting, and 13 included patients from more than one setting (e.g., ward, ICU, or ED). The recruitment periods ranged from one day (cross-sectional study) to 20 years (1996–2015). Sample sizes ranged from 58 to 184,875. Some 27 studies reported data on in-hospital mortality and 16 reported data on 1-month mortality (Table 1).

### 3.3. Risk of Bias within Studies

The individual assessments of risk of bias for the individual studies can be found in Appendix H.

“Selection bias” and “bias in definition” were the most common biases. The most noticeable inconsistency between all of the reviewed studies revolved around the definition of qSOFA. “Outcome measurement bias” was the least common bias (Table 2).

#### 3.3.1. Criteria of qSOFA

The original cut-off values for respiratory rate and systolic blood pressure were followed by most studies. There were large disagreements in the definitions of “altered mentation” between different papers. It was variously defined as different levels of the Glasgow Coma Scale (GCS); different levels of the AVPU (Alert, Pain, Voice, Unresponsive) scale, physician/nursing discretion, and even with more than one criterion being used in the same study, e.g., ‘GCS<14 or anything other than alert on the AVPU scale’.

#### 3.3.2. In-Hospital Mortality

**Figure 2:**
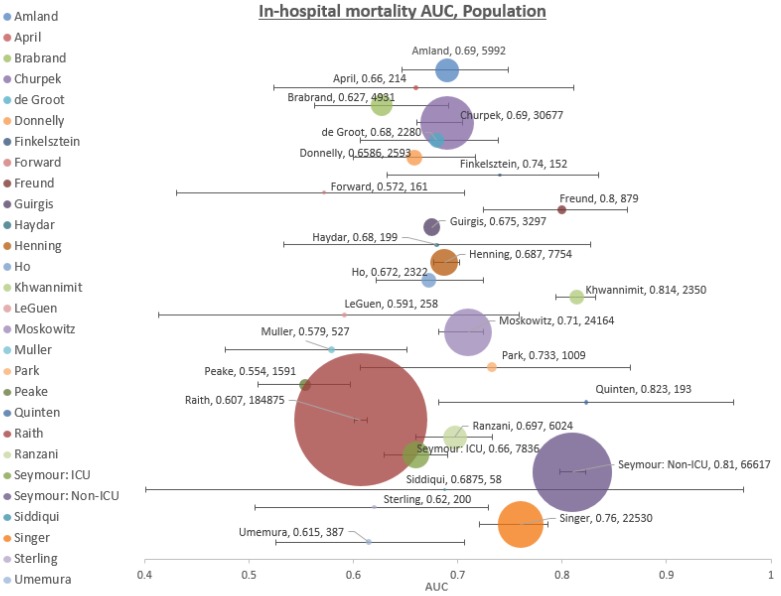
AUROC for in-hospital mortality.

From the 27 studies with a total of 380,041 patients that had data on in-hospital mortality, the median AUROC was 0.68, with a range from 0.55 to 0.82 (Figure 2). A total of 24 studies had data on sensitivity and specificity, ranging from 0.16 to 0.98 (median 0.52) and 0.19 to 0.97 (median 0.81), respectively. Positive and negative predictive values were reported in 18 studies with a range of 0.10–0.38 (median 0.2) and 0.85–0.99 (median 0.95), respectively. Positive and negative likelihood ratios were available in 12 studies, ranging from 1.2 to 4 (median 1.83), and 0.24 to 0.84 (median 0.59), respectively.

A high heterogeneity was confirmed by meta-analysis, with an I^2^ of 98.77%. A meta-analysis would therefore not yield meaningful results, with the data being extracted from these studies.

#### 3.3.3. Month (28/30 Day) Mortality

**Figure 3:**
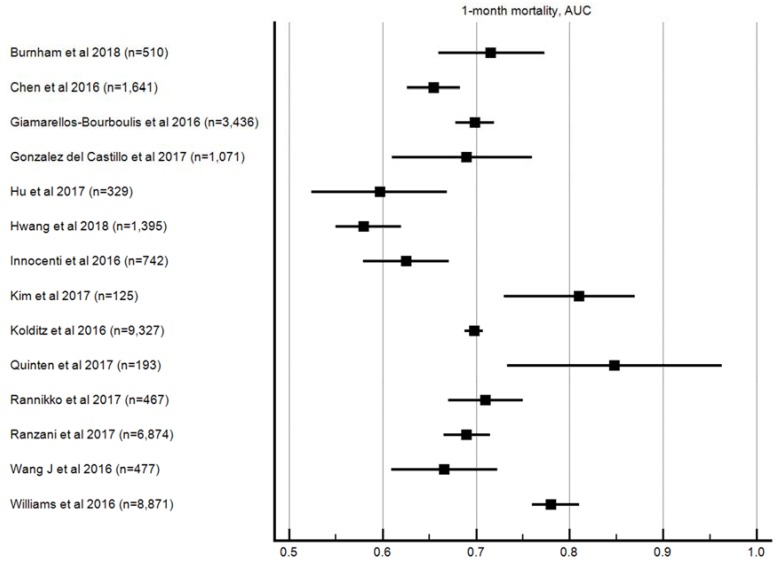
AUROC for 1-month mortality.

A total of 14 studies, with 35,775 patients reported 1-month mortality data (Figure 3). The median AUROC ranged from 0.58 to 0.85 (median 0.69). Sensitivity data were available in 12 of these studies, which ranged from 0.06 to 0.71 (median 0.43); specificity data were available in 13 studies, and ranged from 0.10 to 1.00 (median 0.84). PPV and NPV data were available in 10 studies, and they ranged from 0.14 to 0.68 (median 0.34) and 0.69 to 0.97 (median 0.91), respectively. Positive and negative likelihood ratio data were available in eight studies, and the values ranged from 1.99 to 4.66 (median 2.22) and 0.3 to 0.9 (median 6.43), respectively.

#### 3.3.4. ICU Admission

**Figure 4:**
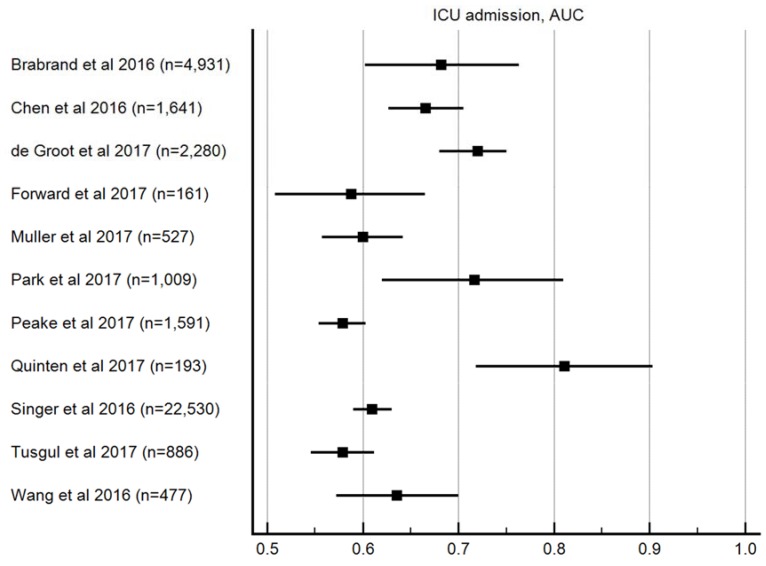
AUROC for ICU admission.

From the 12 studies that reported data on ICU admission, AUROC ranged from 0.58–0.81 (median 0.65, Figure 4. AUROC for ICU admission). Ten studies had data on sensitivity and specificity, which ranged from 0.1 to 0.74 (median 0.37) and 0.42 to 0.97 (median 0.86), respectively. The positive predictive value and negative predictive value data were 0.089–0.578 (median 0.38) in eight studies, and 0.19–0.99 (median 0.90) in nine studies, respectively. Positive and negative likelihood ratio data were available in eight studies, and ranged from 1.27 to 9.97 (median 2.68) and 0.5 to 0.9 (median 0.63), respectively.

#### 3.3.5. Hospital and ICU Length-of-Stay (LOS)

There were no studies that reported on the predicted ability of qSOFA for median ICU or hospital LOS. However, three studies that reported on median ICU LOS. Studies reported results that ranged from 2.9 to 3.1 days. Hospital LOS, presented in median time in qSOFA-positive patients were available in five studies, ranging from 5 to 15 days (a median of nine days).

#### 3.3.6. Diagnosis of Sepsis/Infection

Infective/septic diagnostic predictive values were only presented in two studies, Forward et al. [27] reported an AUROC for patients diagnosed with sepsis to be 0.88, and Brabrand et al. [19] reported an AUROC 0.88 for patients with a diagnosis of infection.

### 3.4. Summary of Results

**Figure A1:**
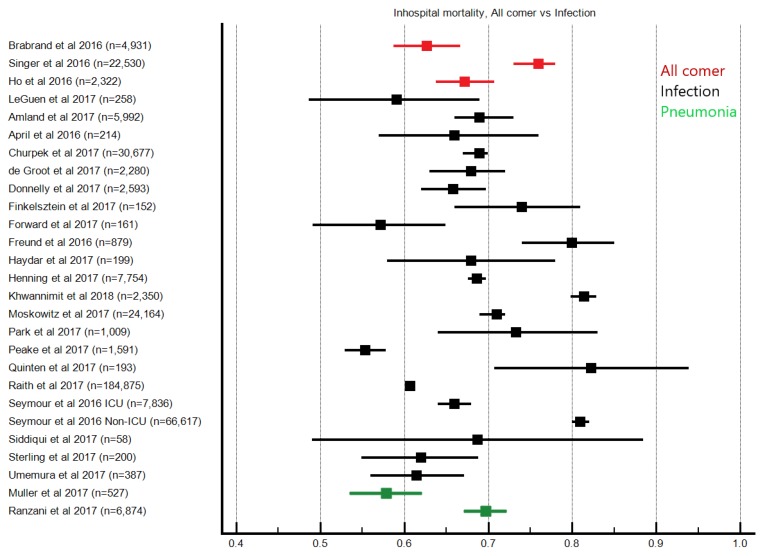
In-hospital Mortality, All Comers vs. Infection.

**Figure A2:**
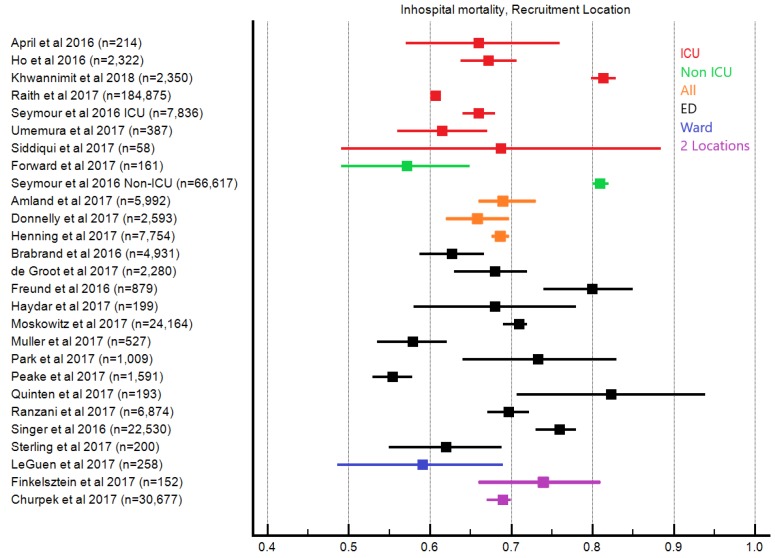
In-hospital Mortality, Recruitment Location.

**Figure A3:**
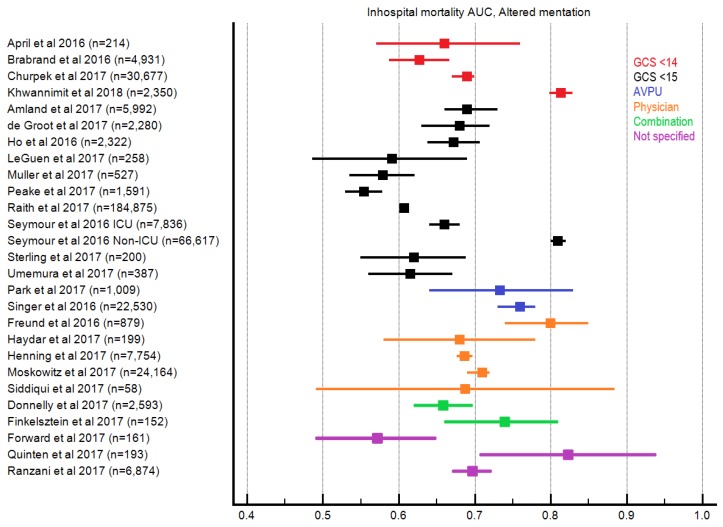
In-hospital Mortality, Altered Mentation.

**Figure A4:**
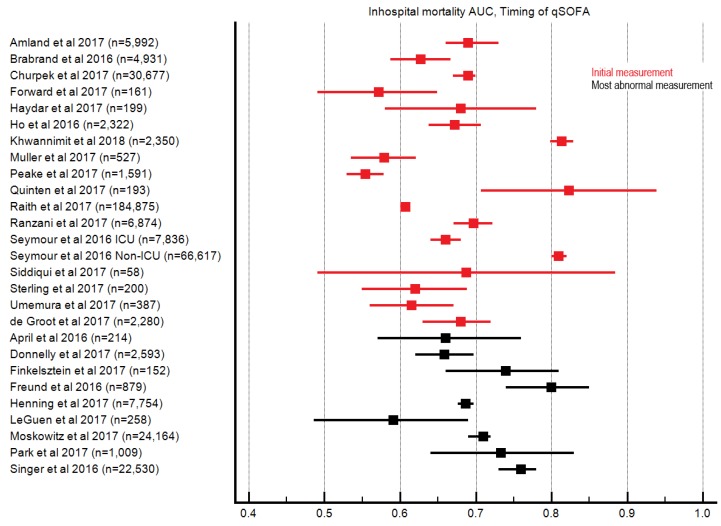
In-hospital mortality, Timing of qSOFA.

Subgroup analyses of AUROC of in-hospital mortality were inconclusive. There was no obvious difference between location of patients who presented with or without infection (Appendix I/Figure A1), location of recruitment/data collection (Appendix J/Figure A2), how mentation was defined or measured (Appendix K/Figure A3), or the timing of qSOFA (Appendix L/Figure A4). A summary of the prognostic values reported from the studies reviewed may be found in Table 3.

## 4. Discussion

This systematic review of 45 studies with 413,634 patients showed that the AUROC of qSOFA for the in-hospital mortality in all patients (with or without suspected infection) was poor, and it showed that it was not suitable for routine clinical use. The AUROC values for other outcomes were also too low for qSOFA to be clinically useful.

qSOFA was developed to predict the likelihood of organ dysfunction in patients with suspected infection [50]. However, the detection of sepsis or infection may be clinically difficult, as symptoms of infection are highly variable [60], and they often mimic other diseases [61]. Misdiagnosis or late diagnosis have been associated with poorer outcomes [62]. Since diagnosis and detection may be difficult to achieve, screening for all patients and not just those with suspected infection would reduce subjectivity and avoidable error in the diagnostic process, and may be a better approach to reduce more severe outcomes and preventable deaths.

When initially introduced, qSOFA was reported to have an AUROC of 0.81 for predicting 1-month mortality. However, this value “was derived from models that include baseline variables plus candidate criteria” [50]. The candidate variables were age, Charlson comorbidity index, race/ethnicity, and gender. A subsequent comparison of the adjusted and unadjusted results in other studies showed that there were substantial differences between the two: Donnelly et al. adjusted 0.76 vs. unadjusted 0.66 [24]; Raith et al. adjusted 0.76 vs. unadjusted 0.61 [48]. We would therefore argue that the adjusted AUROC value reported by the original group bears little relevance for front-line clinicians.

Presenting prognostic predictions using AUROC has limitations [63], as it may be useful on a population scale, but it may not help clinicians on an individual level. In the emergency setting, high sensitivity is particularly important for supporting decisions for triage placement, and for screening and discharging patients; whereas specificity might be more relevant to the ward or ICU setting, to indicate whether a patient’s treatment should be escalated. The data obtained in this review showed the poor sensitivity and mediocre specificity of qSOFA for in-hospital mortality, 1-month mortality, and ICU admission. This suggests qSOFA’s poor utility for screening patients, and its modest value for escalation of care. The positive predictive values were also poor. Although the negative predictive values appeared to be good, the high negative predictive value is likely to reflect on the low incidence of the outcome measure.

The principal idea behind the development of qSOFA was to improve on the pre-existing Systemic Inflammatory Response Syndrome (SIRS) criteria for sepsis identification. Most studies that we reviewed showed that the AUROC for qSOFA outperforms SIRS for predicting in-hospital mortality. However, other scores such as the National Early Warning Score and the Modified Early Warning Score had been reported to have better prognostic values than both SIRS or qSOFA (NEWS 0.77, MEWS 0.73, qSOFA 0.69, and SIRS 0.65) [22]. All three scores had a higher sensitivity at their recommended cut off value when compared to qSOFA (SIRS 0.94, NEWS 0.86, MEWS 0.71, and qSOFA 0.69) [22]. Other systematic reviews focused on the comparison of qSOFA and SIRS, and on qSOFA as a prognostic tool in patients with suspected infection outside of ICU. All three reviews unanimously reported qSOFA’s poor sensitivity [64,65,66].

Two of the three variables in qSOFA are often measured and documented routinely. An assessment of mentation, however, requires experience and clinical judgment. The disagreements in the definition of “altered mentation” were a major source of bias, as they varied between different studies. In Seymour’s original qSOFA paper, the group reported that “the predictive validity of qSOFA was not significantly different when using … the GCS score <15 (*p* = 0.56), compared with the model with GCS score ≤13.” A standardized definition is required for future studies, and details must be added, to further elaborate on how altered mentation is determined in patients with impaired mental status at baseline, e.g., dementia sufferers. This is significant, as infection and sepsis are common causes of delirium in the older population.

The strengths of this review include the large number of study subjects, the inclusive search strategy, and bias assessment from multiple reviewers. However, there are also limitations to our review. We had taken a pragmatic approach in utilizing the qSOFA score, and we have used it on all-comers, rather than only on those with a suspected infection. Changes in treatment outcomes of sepsis made older studies difficult to compare directly with the more recent ones. The small number of prospective studies also limits the validity and generalizability of the results. There were only three prospective studies among the papers reviewed.

## 5. Conclusions

In conclusion, our group found that qSOFA is not a clinically useful prognostic tool for in-hospital, 1-month mortality, or ICU admission for all-comers, with or without suspected infection.

## Figures and Tables

**Table 1:** Characteristics of the studies included in the systematic review of qSOFA for predicting prognosis.

Study	Median Age (IQR) Mean Age ± SD	Location	Male (%)	Sample Size	Study Type	Recruitment Period
Amland et al. [15]	65 (51–76)	US	48	5992	Retrospective	January 2016–March 2016
April et al. [16]	72 (60–79)	Texas, US	58.9	214	Retrospective	August 2012–February 2015
Askim et al. [17]	62 (41–78)	Norway	53	1535	PCDRC	January 2012–December 2012
Boulos et al. [18]	68.5 ± 17.4	Monash, Australia	52	646	Retrospective	January 2015–December 2015
Brabrand et al. [19]	65 (50–77)	Denmark	49.2	4931	Retrospective	October 2008–May 2010
Burnham et al. [20]	61.1 (51.6–69.8)	Missouri, USA	52	510	Retrospective	June 2009–December 2013
Chen et al. [21]	73 (62–79)	Beijing, China	59	1641	PCDRC	January 2012–May 2014
Churpek et al. [22]	58 ± 18	Chicago, US	47	30,677	Retrospective	November 2008–January 2016
de Groot et al. [23]	61.1 ± 17	Holland	57.7	2280	PCDRC	April 2011–February 2016
Donnelly et al. [24]	68 (61–75)	USA	47.8	2593	Retrospective	January 2003–October 2007
Du et al. [25]	56.4 ± 18.1	Sichuan, China	65.7	565	Retrospective	August 2015–July 2016
Finkelsztein et al. [26]	64 (51–75)	New York, USA	31	152	PCDRC	October 2014–July 2016
Forward et al. [27]	70 ± ?	Sydney, Australia	55	161	Prospective	May 2015–August 2015
Freund et al. [28]	67 (48–81)	Europe	53	879	Prospective	May 2016–June 2016
Giamarellos-Bourboulis et al. [29]	76 (IQR: 22)	Greece	?	3436	Retrospective	May 2006–December 2015
Gonzalez del Castillo et al. [30]	83.6 ± 5.6	Spain	50.8	1071	Prospective	October 2015–April 2016
Guirgis et al. [31]	59 (48–70)	Florida, USA	49	3297	Retrospective	October 2013–May 2016
Haydar et al. [32]	71 (range 18–102)	Portland, USA	55	199	Retrospective	September 2014–September 2015
Henning et al. [33]	58.4 ± 20.1	USA	52.2	7754	PCDRC	December 2003–September 2006
Ho et al. [34]	57.1 (41–70)	Perth, Australia	61	2322	PCDRC	January 2008–December 2013
Hu et al. [35]	?	Zhejiang, China	62.6	329	Retrospective	January 2015–June 2015
Hwang et al. [36]	65 (55–73)	Seoul, South Korea	56	1395	Retrospective	August 2008–September 2014
Innocenti et al. [37]	75 ± 14	Florence, Italy	53	742	Retrospective	June 2008–April 2016
Khwannimit et al. [38]	62 (45–75)	Songkhla, Thailand	56.1	2350	Retrospective	January 2007–December 2016
Kim et al. [39]	72 (59.5–80)	Seoul, South Korea	62.4	125	Retrospective	January 2014–December 2014
Kolditz et al. [40]	63 (?)	Germany	56	9327	Retrospective	October 2002–June 2015
LeGuen et al. [41]	72 (57–82)	Victoria, Australia	48	258	Prospective	6 June 2016, 10 July 2016
Moskowitz et al. [42]	63.8 ± 18.1	USA	50.9	24,164	Retrospective	January 2010–December 2014
Muller et al. [43]	66 (50–76)	Switzerland	64.5	527	Retrospective	June 2011–May 2013
Park et al. [44]	67.4 ± 17.6	Seoul, South Korea	45	1009	Retrospective	March 2007–February 2016
Peake et al. [45]	62.9 ± 16.5	Australasia	59.7	1591	PCDRC	October 2008–April 2014
Quinten et al. [46]	60 (48–71)	Netherlands	56	193	PCDRC	August 2012–April 2014
Raith et al. [47]	62.9 ± 17.4	Australasia	55.4	184,875	Retrospective	January 2000–December 2015
Rannikko et al. [48]	68 (58–78)	Finland	53	467	Retrospective	March 2012–February 2014
Ranzani et al. [49]	66.1 ± 19	Barcelona + Valencia, Spain	62.2	6874	PCDRC	January 1996–December 2015
Seymour et al. [50]	61 ± 19	US and Germany	43	74,453	Retrospective	January 2010–December 2012
Siddiqui et al. [51]	64.4 ± 12.9	Singapore	60	58	Retrospective	January 2015–December 2015
Singer et al. [52]	54 ± 21	New York, USA	47	200	Retrospective	January 2014–March 2015
Sterling et al. [53]	60 ± 16.7	USA	?	22,530	PCDRC	August 2004–January 2009
Szakmany et al. [54]	74 (61–83)	Wales, UK	47	380	Prospective	19 October 2016
Tusgul et al. [55]	80 (69–87)	Switzerland	52.1	886	Retrospective	January 2012–December 2012
Umemura et al. [56]	?	Japan	59.7	387	PCDRC	June 2010–May 2011
Wang J et al. [57]	73 (60–79)	Beijing, China	61.8	477	PCDRC	July 2015–December 2015
Wang S et al. [58]	63 ± 17.3	Chenzhou, China	69.5	311	Retrospective	July 2012–June 2016
Williams et al. [59]	49 (30–69)	Brisbane, Australia	51.3	8871	PCDRC	October 2007–May 2011

qSOFA, quick Sepsis-related Organ Failure Assessment; IQR, Interquartile Range; PCDRC, Prospectively Collected Data Retrospective Cohort; ?, Information not available.

**Table 2:** Risk of bias across the studies.

Author Year	Selection Bias	Bias in Definition and Measurement	Outcome Measurement Bias	Handling of Missing Data	Confounding	Bias of Statistics or Presentation of Result
Amland et al. 2017						
April et al. 2016						
Askim et al. 2017						
Boulos et al. 2017						
Brabrand et al. 2016						
Burnham et al. 2018						
Chen et al. 2016						
Churpek et al. 2017						
de Groot et al. 2017						
Donnelly et al. 2017						
Du et al. 2017						
Finkelsztein et al. 2017						
Forward et al. 2017						
Freund et al. 2016						
Giamarellos-Bourboulis et al. 2016						
Gonzalez del Castillo et al. 2017						
Guirgis et al. 2017						
Haydar et al. 2017						
Henning et al. 2017						
Ho et al. 2016						
Hu et al. 2017						
Hwang et al. 2018						
Innocenti et al. 2016						
Khwannimit et al. 2018						
Kim et al. 2017						
Kolditz et al. 2016						
LeGuen et al. 2017						
Moskowitz et al. 2017						
Muller et al. 2017						
Park et al. 2017						
Peake et al. 2017						
Quinten et al. 2017						
Raith et al. 2017						
Rannikko et al. 2017						
Ranzani et al. 2017						
Seymour et al. 2016						
Siddiqui et al. 2017						
Singer et al. 2016						
Sterling et al. 2017						
Szakmany et al. 2018						
Tusgul et al. 2017						
Umemura et al. 2017						
Wang J et al. 2016						
Wang S et al. 2017						
Williams et al. 2016						

Green, low risk; Yellow, moderate risk; Red, high risk.

**Table 3:** Summary of the prognostic values reported from the studies reviewed.

	qSOFA Median Value Min–Max (Number of Patients that the Value is Derived from)						
Outcomes	AUROC	Sensitivity	Specificity	PPV	NPV	LR+	LR−
In-hospital mortality	0.68	0.52	0.81	0.2	0.94	1.83	0.59
	0.55–0.82	0.16–0.98	0.19–0.97	0.07–0.38	0.85–0.99	1.15–4	0.24–0.84
	(*n* = 380,920)	(*n* = 118,051)	(*n* = 118,051)	(*n* = 67,555)	(*n* = 90,085)	(*n* = 24,925)	(*n* = 24,925)
1-month mortality	0.69	0.43	0.84	0.34	0.91	2.22	6.43
	0.58–0.85	0.06–0.71	0.10–1.00	0.14–0.68	0.69–0.97	1.26–3.71	2.17–14.4
	(*n* = 36,415)	(*n* = 34,462)	(*n* = 36,415)	(*n* = 26,603)	(*n* = 26,603)	(*n* = 8121)	(*n* = 8121)
ICU admission	0.65	0.37	0.86	0.38	0.9	2.68	0.63
	0.58–0.81	0.1–0.74	0.42–0.97	0.09–0.90	0.19–0.99	1.27–9.97	0.5–0.9
	(*n* = 37,105)	(*n* = 33,816)	(*n* = 33,816)	(*n* = 11,093)	(*n* = 33,623)	(*n* = 11,286)	(*n* = 11,286)

qSOFA, quick Sepsis-related Organ Failure Assessment; AUROC, Area Under the Receiver Operating Characteristics curve; PPV, Positive Predicted Value; NPV, Negative Predicted Value; LR+, Positive Likelihood Ratio; LR−, Negative Likelihood Ratio; ICU, Intensive Care Unit.

## Appendix A. Articles in Non-English Languages

German articles:

[67] Christ, M.; Geier, F.; Bertsch, T.; Singler, K. Sepsis in emergency medicine. *Dtsch. Med. Wochenschr.* **2016**, *141*, 1074.

[68] Dickmann, P.; Scherag, A.; Coldewey, S.M.; Sponholz, C.; Brunkhorst, FM.; Bauer, M. Epistemology in the intensive care unit—What is the purpose of a definition?: Paradigm shift in sepsis research. *Der Anaesth.* **2017**, *66*, 622–625.

[69] Leidel, B.A. The new Sepsis 3 definition—Flop or top? *Notf. Rettungsmed.* **2017**, *20*, 383.

[70] Gerlach, J. The new Sepsis 3 definition—A courageous approach. *Notf. Rettungsmed.* **2017**, *20*, 385–389.

Spanish article:

[71] Julián-Jiménez, A.; Yañez, M.C.; del Castillo, J.G.; Salido-Mota, M.; Mora-Ordoñez, B.; Arranz-Nieto, M.J.; Chanovas-Borras, M.R.; Llopis-Roca, F.; Mòdol-Deltell, J.M.; Muñoz, G. Poder pronóstico de mortalidad a corto plazo de los biomarcadores en los ancianos atendidos en Urgencias por infección. *Enferm. Infecci. Microbiol. Clín.* **2017**.

Russian article:

[72] Lebedev, N.V.; Klimov, A.E.; Agrba, S.B.; Gaidukevich, E.K. Combined forecasting system of peritonitis outcome. *Khirurgiia* **2017**, *9*, 33–37.

French article:

[73] Lemachatti, N.; Freund, Y. Sepsis: définitions et validations. *Ann. Fr. Méd. D’urgence* **2017**, *7*, 30–34.

## Appendix B. OVID Search Strategy

1. qSOFA.mp.
2. quick SOFA.mp.
3. quick sequential organ failure assessment.mp.
4. quick sepsis-related organ failure assessment.mp.
5. 1 or 2 or 3 or 4
6. mortality.mp.
7. 5 and 6

## Appendix C. WHO International Clinical Trails Registry Platform

qSOFA OR quick SOFA OR quick sequential organ failure assessment OR quick sepsis-related organ failure assessment AND Mortality.

## Appendix D. Web of Science

TOPIC: (qSOFA OR quick SOFA OR quick sequential organ failure assessment OR quick sepsis-related organ failure assessment) AND TOPIC: (mortality) Timespan: All years. Indexes: SCI-EXPANDED, SSCI, A&HCI, CPCI-S, CPCI-SSH, BKCI-S, BKCI-SSH, ESC.

## Appendix E. Scopus

ALL ((qsofa OR quick AND sofa OR quick AND sequential AND organ AND failure AND assessment OR quick AND sepsis-related AND organ AND failure AND assessment) AND mortality).

## Appendix F. ClinicalTrials.gov

(qSOFA OR quick SOFA OR quick sequential organ failure assessment OR quick sepsis-related organ failure assessment) AND Mortality.

## Appendix G. Studies Excluded

**Table A1:** Studies Excluded.

Author	Title	Decisions
Andaluz, D., Ferrer, R.	SIRS, qSOFA, and organ failure for assessing sepsis at the emergency department.	Excluded, no original data
April, M.D., Lantry, J.H.	Prognostic Accuracy of Quick Sequential Organ Failure Assessment Among Emergency Department Patients Admitted to an ICU.	Excluded, no original data
Asai, N., Watanabe, H., Shiota, A., et al.	Could qSOFA and SOFA score be correctly estimating the severity of healthcare-associated pneumonia?	Excluded, no original data
Atalan, H.K., Güçyetmez, B.	The effects of the chloride:sodium ratio on acid–base status and mortality in septic patients	Excluded, Study aim irrelevant
Awad, A. Bader-El-Den, M., McNicholas, J., et al.	Early hospital mortality prediction of intensive care unit patients using an ensemble learning approach.	Excluded, Study aim irrelevant
Becchi, C., Al Malyan, M., Fabbri, L.P., et al.	Mean platelet volume trend in sepsis: Is it a useful parameter? [Andamento del volume piastrinico medio in sepsi: Un parametro utile?]	Excluded, Study aim irrelevant
Bhattacharjee, P., Edelson, D.P., Churpek, M.M.	Identifying Patients with Sepsis on the Hospital Wards.	Excluded, no original data
Biyikli, E., Kayipmaz, A.E., Kavalci, C.	Effect of platelet–lymphocyte ratio and lactate levels obtained on mortality with sepsis and septic shock.	Excluded, Study aim irrelevant
Busani, S., Girardis, M.	PSP/reg: A new stone in sepsis biomarkers?	Excluded, Study aim irrelevant
Christ, M., Geier, F., Bertsch, T., et al.	Sepsis in Emergency Medicine. [German]	Language German
Cour, M., Hernu, R., Bénet, T., et al.	Benefits of smart pumps for automated changeovers of vasoactive drug infusion pumps: A quasi-experimental study	Excluded, Study aim irrelevant
David, N., Roux, N., Clavier, E., et al.	Open repair of extensive thoracoabdominal and thoracic aneurysm: A preliminary single-center experience with femorofemoral distal aortic perfusion with oxygenator and without cerebrospinal fluid drainage	Excluded, Study aim irrelevant
Desautels, T., Calvert, J., Hoffman, J., et al.	Prediction of Sepsis in the Intensive Care Unit with Minimal Electronic Health Record Data: A Machine Learning Approach.	Excluded, Study aim irrelevant
Dickmann, P., Scherag, A., Coldewey, S.M., et al.	Epistemology in the intensive care unit—What is the purpose of a definition? Paradigm shift in sepsis research	Language German
Du, B., Weng, L.	Systemic inflammatory response syndrome, sequential organ failure assessment, and quick sequential organ failure assessment: More pieces needed in the sepsis puzzle	Excluded, no original data
Edmark, C., McPhail, M.J.W., Bell, M., et al.	LiFe: A liver injury score to predict outcome in critically ill patients	Excluded, Study aim irrelevant
Fukushima, H., Kobayashi, M., Kawano, K., et al.	Performance of qSOFA and SOFA for predicting mortality in patients with acute pyelonephritis associated with upper urinary tract calculi.	Excluded. Patient too specific
Gerlach, H.	The new Sepsis 3 definition—a courageous approach	Language German
del Castillo, J.G., Carlota, C., Candel, F.J., et al.	New sepsis criteria: do they replace or complement what is known in the approach to the infectious patient?	Excluded, no original data
Gul, F., Arslantas, M.K., Cinel, I., et al.	Changing Definitions of Sepsis. [Review]	Excluded, no original data
Hou, P.C., Seethala, R.R., Aisiku, I.P.	qSOFA—Welcome to the sepsis alphabet soup	Excluded, no original data
Huson, M.A., Kalkman, R., Grobusch, M.P., et al.	Predictive value of the qSOFA score in patients with suspected infection in a resource limited setting in Gabon.	Excluded. Patient too specific
Huson, M.A.M., Katete, C., Chunda, L., et al.	Application of the qSOFA score to predict mortality in patients with suspected infection in a resource-limited setting in Malawi.	Excluded. Patient too specific
Jacob, J.A.	New sepsis diagnostic guidelines shift focus to organ dysfunction.	Excluded, no original data
Jawa, R.S., Vosswinkel, J.A., McCormack, J.E., et al.	Risk assessment of the blunt trauma victim: The role of the quick Sequential Organ Failure Assessment Score (qSOFA).	Excluded. Patient too specific
Julian-Jimenez, A., Yanez, M.C., Gonzalez-del Castillo, J., et al.	Prognostic power of biomarkers for short-term mortality in the elderly patients seen in Emergency Departments due to infections. [Spanish]	Language Spanish
Kim, M., Ahn, S., Kim, W.Y., et al.	Predictive performance of the quick Sequential Organ Failure Assessment score as a screening tool for sepsis, mortality, and intensive care unit admission in patients with febrile neutropenia.	Excluded. Patient too specific
Ladhani, H.A., Sajankila, N., Zosa, B.M., et al.	Utility of Sequential Organ Failure Assessment score in predicting bacteremia in critically ill burn patients.	Excluded. Patient too specific
Lebedev, N.V., Klimov, A.E., Agrba, S.B., et al.	[Combined forecasting system of peritonitis outcome]. [Russian]	Language Russian
Leclerc, F., Duhamel, A., Deken, V., et al.	Can the pediatric logistic organ dysfunction-2 score on day 1 be used in clinical criteria for sepsis in children?	Excluded. Patient too specific
Lee, S.J., Ramar, K., Park, J.G., et al.	Increased fluid administration in the first three hours of sepsis resuscitation is associated with reduced mortality: A retrospective cohort study	Excluded, Study aim irrelevant
Leidel, B.A.	The new Sepsis 3 definition—Flop or top?	Language German
Lemachatti, N., Freund, Y.	Sepsis: Definitions and validations. [French]	Language French
Maegele, M., Lefering, R., Yucel, N., et al.	Early coagulopathy in multiple injury: An analysis from the German Trauma Registry on 8724 patients	Excluded, Study aim irrelevant
Marik, P.E., Taeb, A.M.	SIRS, qSOFA, and new sepsis definition	Excluded, no original data
McCormack, D., Kulkarni, M., Keller, S.E.	Perspectives and implications of the new sepsis clinical practice guidelines.	Excluded, no original data
McLymont, N., Glover, G.W.	Scoring systems for the characterization of sepsis and associated outcomes.	Excluded, no original data
Moore, C.C., Hazard, R., Saulters, K.J., et al.	Derivation and validation of a universal vital assessment (UVA) score: a tool for predicting mortality in adult hospitalised patients in sub-Saharan Africa.	Excluded. Patient too specific
Patidar, K.R., Shaw, J., Acharya, C., et al.	No Association Between Quick Sequential Organ Failure Assessment and Outcomes of Patients With Cirrhosis and Infections.	Excluded. Patient too specific
Peach, BC.	Implications of the new sepsis definition on research and practice.	Excluded, no original data
Piano, S., Bartoletti, M., Tonon, M., et al.	Assessment of Sepsis-3 criteria and quick SOFA in patients with cirrhosis and bacterial infections.	Excluded. Patient too specific
Rasulo, F.A., Bellelli, G., Ely, E.W., et al.	Are you Ernest Shackleton, the polar explorer? Refining the criteria for delirium and brain dysfunction in sepsis	Excluded, no original data
Rhee, C., Klompas, M.	New Sepsis and Septic Shock Definitions Clinical Implications and Controversies	Excluded, no original data
Ronco, C., Legrand, M., Goldstein, S.L., et al.	Neutrophil gelatinase-associated lipocalin: Ready for routine clinical use? An international perspective	Excluded, no original data
Rothman, M., Levy, M., Dellinger, R.P., et al.	Sepsis as 2 problems: Identifying sepsis at admission and predicting onset in the hospital using an electronic medical record-based acuity score	Excluded, Study aim irrelevant
Sager, R., Wirz, Y., Amin, D., et al.	Are admission procalcitonin levels universal mortality predictors across different medical emergency patient populations? Results from the multi-national, prospective, observational TRIAGE study.	Excluded, Study aim irrelevant
Scheer, C.S., Kuhn, S.O., Rehberg, S.	Use of the qSOFA score in the emergency department.	Excluded, no original data
Schlapbach, L.J., Straney, L., Bellomo, R., et al.	Prognostic accuracy of age-adapted SOFA, SIRS, PELOD-2, and qSOFA for in-hospital mortality among children with suspected infection admitted to the intensive care unit.	Excluded. Patient too specific
Scott, M.C.	Defining and Diagnosing Sepsis.	Excluded, no original data
Seckel, M.A.	Sepsis-3: The new definitions.	Excluded, no original data
Seckel, M.A., Ahrens, T.	Challenges in Sepsis Care: New Sepsis Definitions and Fluid Resuscitation Beyond the Central Venous Pressure.	Excluded, no original data
Serafim, R., Gomes, J.A., Salluh, J., et al.	A comparison of the quick-SOFA (qSOFA) and SIRS criteria for the diagnosis of sepsis and prediction of mortality: A systematic review and meta-analysis.	Excluded, no original data
Shetty, A., MacDonald, S.P., Williams, J.M., et al.	Lactate ≥ 2 mmol/L plus qSOFA improves utility over qSOFA alone in emergency department patients presenting with suspected sepsis.	Excluded, Study aim irrelevant
Singer, M., Deutschman, C.S., Seymour, C., et al.	The third international consensus definitions for sepsis and septic shock (sepsis-3).	Excluded, no original data
Solligard, E., Damas, J.K.	SOFA criteria predict infection-related in-hospital mortality in ICU patients better than SIRS criteria and the qSOFA score.	Excluded, no original data
Viale, P., Tedeschi, S., Scudeller, L., et al.	Infectious diseases team for the early management of severe sepsis and septic shock in the emergency department	Excluded, Study aim irrelevant
Vincent, J.L., Grimaldi, D.	Quick sequential organ failure assessment: Big databases vs. intelligent doctors.	Excluded, no original data
Wang, A.Y., Ma, H.P., Kao, W.F., et al.	Red blood cell distribution width is associated with mortality in elderly patients with sepsis.	Excluded, Study aim irrelevant
Wang, H.E., Jones, A.R., Donnelly, J.P.	Revised National Estimates of Emergency Department Visits for Sepsis in the United States	Excluded, Study aim irrelevant
Zaccone, V., Tosoni, A., Passaro, G., et al.	Sepsis in Internal Medicine wards: Current knowledge, uncertainties and new approaches for management optimization.	Excluded, no original data
Zhou, X., Ding, B., Ye, Y., Tang, G., et al.	Authors respond to Both qSOFA score and bedside plasma lactate are the predictors of mortality for patients with infections in ED.	Excluded, no original data
Zhou, X., Tang, G.	Quick sepsis-related organ failure assessment (qSOFA) predicting outcomes in patients with infection, some lingering doubts.	Excluded, no original data
Zhou, X.D., Zhang, J.Y., Liu, W.Y., et al.	Quick chronic liver failure-sequential organ failure assessment: An easy-to-use scoring model for predicting mortality risk in critically ill cirrhosis patients	Excluded. Patient too specific

## Appendix H. Characteristics of Studies

**First Author (Year)**	**Amland RC (2017) [15]**
Title	Quick Sequential [Sepsis-Related] Organ Failure Assessment (qSOFA) and St. John Sepsis Surveillance Agent to Detect Patients at Risk of Sepsis: An Observational Cohort Study.
Journal	American Journal of Medical Quality
Reviewer	RL, MB, LL
Study sponsor	Nil
Study type	Multi-centered retrospective cohort (January–March 2016)
Location	United States
Participants Number Male/Female Median age Patient group	5992 48% male 65 (51–76) Hospitalized adults with suspected infection, defined in Sepsis-3
qSOFA criteria	Respiratory rate ≥22 bpm, systolic blood pressure ≤100 mmHg, and Glasgow Coma Score (GCS) <15
Primary outcome Other outcomes	In-hospital mortality Composite of death or ICU admission
Results	In-hospital mortality AUC 0.69 (95% CI 0.66 to 0.73)
Note	

**Risk of Bias**	**Author’s Judgment** **Low Risk** **Unclear** **High Risk**	**Support for Judgment**
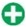
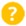
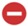
Selection bias		
Bias in definition and measurement		Definition of sepsis is chart-based
Outcome measurement bias		
Handling of missing data		Not mentioned
Confounding		Retrospective
Bias of statistics or presentation of result		Possible double counting in modelling

**First Author (Year)**	**April MD (2016) [16]**
Title	Sepsis clinical criteria in emergency department patients admitted to an intensive care unit: An external validation study of quick sequential organ failure assessment
Journal	The Journal of Emergency Medicine
Reviewer	RL, KH, LL, MB, CG
Study sponsor	No information given
Study type	Retrospective cohort (August 2012–February 2015)
Location	Texas, USA
Participants Number Male/Female Median age (IQR) Patient group	321 identified, 214 analyzed 58.9% male 72 (60–79) ICU admission from ED with presumed sepsis; Patient with non-infectious etiology excluded
qSOFA criteria	Respiratory rate > 22 breaths/min; Glasgow Coma Scale < 14; Systolic blood pressure < 100 mm Hg
Primary outcome Other outcomes	Prognostic accuracy of qSOFA and SIRS for predicting in-hospital mortality (AUROC, sensitivity, specificity, and likelihood ratio) Assessment of the prognostic accuracy of LODS and SOFA criteria, using the same measures
Results	0.66 (95% CI 0.57–0.76) for qSOFA, 89.7% sensitivity, 27.4% specificity, 1.2 positive likelihood ratio, and 0.4 negative likelihood ratio

**Risk of Bias**	**Author’s Judgment** **Low Risk** **Unclear** **High Risk**	**Support for Judgment**
Selection bias		Only ICU patients involved; Selective patients
Bias in definition and measurement		RR > 22 breaths/min; sBP < 100; Altered mentation: GCS < 14
Outcome measurement bias		
Handling of missing data		Not explicit
Confounding		
Bias of statistics or presentation of result		Potential presentation error in Table 3; No selective reporting of results

**First Author (Year)**	**Askim A (2017) [17]**
Title	Poor performance of quick-SOFA (qSOFA) score in predicting severe sepsis and mortality—A prospective study of patients admitted with infection to the emergency department.
Journal	Scandinavian Journal of Trauma, Resuscitation & Emergency Medicine
Reviewer	RL, CG, MB
Study sponsor	Central Norway Regional Health Authority (RHA) and the NorwegianUniversity of Science and Technology (NTNU), Trondheim Norway.
Study type	Prospectively Collected Data Retrospective Cohort (January–Decemeber 2012)
Location	Norway
Participants Number Male/Female Median age Patient group	1535 53% male 62 (41–78) All patients with suspected or confirmed infection
qSOFA criteria	Respiratory rate ≥ 22 bpm, systolic blood pressure ≤ 100 mmHg, and Glasgow Coma Score (GCS) < 15
Primary outcome Other outcomes	?
Results	qSOFA ≥2 Sensitivity 0.13 (0.05–0.25) Specificity 0.96 (0.95–0.97) PPV 0.14 (0.07–0.23) NPV 0.96 (0.96–0.96)
Note	16 years old and older

**Risk of Bias**	**Author’s Judgment** **Low Risk** **Unclear** **High Risk**	**Support for Judgment**
Selection bias		
Bias in definition and measurement		Sepsis defined by SIRS criteria
Outcome measurement bias		
Handling of missing data		10% missing data
Confounding		
Bias of statistics or presentation of result		

**First Author (Year)**	**Boulos D (2017) [18]**
Title	Predictive value of quick Sepsis-Related Organ Failure Scores following sepsis-related Medical Emergency Team calls: A retrospective cohort study
Journal	Anesthetic Intensive Care
Reviewer	RL, CG, MB
Study sponsor	Nil noted
Study type	Retrospective cohort (January 2015–Decemeber 2015)
Location	Monash Health, Australia
Participants Number Male/Female Median age Patient group	646 52% male 68.52 ± 17.4 (mean) Patients who had sepsis-related Medical Emergency Team calls
qSOFA criteria	Not defined
Primary outcome Other outcomes	28-day, in-hospital mortality ICU admission, need for inotropic or ventilatory support, made not-for-resuscitation, repeat Medical Emergency Team (MET) call
Results	28-day mortality AUC 0.64 for qSOFA
Note	

**Risk of Bias**	**Author’s Judgment** **Low Risk** **Unclear** **High Risk**	**Support for Judgment**
Selection bias		Ward patients with MET calls only
Bias in definition and measurement		SIRS to define sepsis
Outcome measurement bias		
Handling of missing data		Not reported/ Not mentioned
Confounding		Could not be assessed
Bias of statistics or presentation of result		

**First Author (Year)**	**Brabrand M (2016) [19]**
Title	Validation of the qSOFA score for identification of septic patients: A retrospective study
Journal	European Journal of Internal Medicine
Reviewer	RL, KH, LL, MB, CG
Study sponsor	No external funding
Study type	Retrospective cohort (Letter)
Location	Denmark
Participants Number Male/Female Median age (IQR) Patient group	4931 analyzed 49.2% male 65 (50–77) ED patients who are acutely admitted under medicine
qSOFA criteria	RR greater or equal to 22, sBP lesser or equal to 100, and altered mentation <14
Primary outcome Other outcomes	Hospital mortality and ICU admission Hospital mortality, and ICU admission individually
Results	Hospital mortality AUROC 0.627 (0.587–0.667)
Note	The author of this article is also one of the reviewers of this review article

**Risk of Bias**	**Author’s Judgment** **Low Risk** **Unclear** **High Risk**	**Support for Judgment**
Selection bias		Only medical patients included
Bias in definition and measurement		
Outcome measurement bias		
Handling of missing data		Not stated in paper but asked in person.
Confounding		
Bias of statistics or presentation of result		

**First Author (Year)**	**Burnham JP (2018) [20]**
Title	qSOFA score: Predictive validity in Enterobacteriaceae bloodstream infections.
Journal	Journal of Critical Care
Reviewer	RL, CG, MB
Study sponsor	Nil
Study type	Retrospective cohort (June 2009–Decemeber 2013)
Location	USA
Participants Number Male/Female Median age Patient group	510 52% male 61.1 (51.6–69.8) all patients age ≥ 18 with sepsis, severe sepsis, or septic shock, and a positive blood culture for an organism in the Enterobacteriaceae family
qSOFA criteria	Altered mental status—Reported by family, RR 32(?)
Primary outcome Other outcomes	All-cause 30-day mortality Nil
Results	30-day mortality AUC 0.716 for qSOFA ≥2
Note	Sepsis as defined by systemic inflammatory response syndrome (SIRS) criteria Second analysis

**Risk of Bias**	**Author’s Judgment** **Low Risk** **Unclear** **High Risk**	**Support for Judgment**
Selection bias		Only Enterobacteriaceae
Bias in definition and measurement		AMS not well-defined
Outcome measurement bias		Hospice discharge considered dead
Handling of missing data		Reported missing data, but did not explain how they responded to this
Confounding		Young patients and large Afro-American population
Bias of statistics or presentation of result		

**First Author (Year)**	**Chen YX (2016) [21]**
Title	Use of CRB-65 and quick Sepsis-related Organ Failure Assessment to predict site of care and mortality in pneumonia patients in the emergency department: A retrospective study
Journal	Critical Care
Reviewer	RL, KH, LL, MB, CG
Study sponsor	No information provided
Study type	Prospectively Collected Data Retrospective Cohort (January 2012–May 2014)
Location	Beijing, China
Participants Number Male/Female Median age (IQR) Patient group	1769 identified, 1641 analyzed 59% male 73 (62–79) ED patients with new infiltrates on chest radiograph and two or more symptoms consistent with pneumonia (including cough, dyspnea, fever, sputum production, breathlessness, and/or pleuritic chest pain)
qSOFA criteria	Respiratory rate ≥22/minute, altered mentation (Glasgow Coma Scale score ≤13) and systolic blood pressure ≤100 mmHg.
Primary outcome Other outcomes	All-cause mortality at 28 days Hospitalization and ICU admission
Results	28 day mortality qSOFA AUC 0.655 (0.626–0.683)
Note	Ethics for current study not stated

**Risk of Bias**	**Author’s Judgment** **Low Risk** **Unclear** **High Risk**	**Support for Judgment**
Selection bias		Restrictive inclusive criteria Small number of sample
Bias in definition and measurement		Cut-off value assumed to be Glasgow Coma Scale ≤13
Outcome measurement bias		
Handling of missing data		
Confounding		Smoking status of patients not included
Bias of statistics or presentation of result		Potential Table 3 error: qSOFA 2 or >2

**First Author (Year)**	**Churpek MM (2017) [22]**
Title	qSOFA, SIRS, and early warning scores for detecting clinical deterioration in infected patients outside the ICU
Journal	American Journal of Respiratory and Critical Care Medicine
Reviewer	RL, KH, LL, MB, CG
Study sponsor	University of Chicago
Study type	Retrospective cohort (November 2008–January 2016)
Location	Chicago, USA
Participants Number Male/Female Age Patient group	150,288 identified, 30,677 analyzed 47% male Mean 58 years old (SD 18.0) All patients (ED and ward) outside of ICU with suspected infection
qSOFA criteria	Systolic blood pressure ≤100 mm Hg, respiratory rate ≥22 breaths per minute, and altered mental status (defined as either a Glasgow Coma Scale score ≤13 or an Alert Voice Pain Unresponsive scale (AVPU) other than “Alert”)
Primary outcome Other outcomes	In-hospital mortality composite of death or ICU stay
Results	In-hospital mortality AUC 0.69 (0.67–0.70)
Note	

**Risk of Bias**	**Author’s Judgment** **Low Risk** **Unclear** **High Risk**	**Support for Judgment**
Selection bias		Definition of sepsis
Bias in definition and measurement		
Outcome measurement bias		
Handling of missing data		66% of admissions were excluded due to missing data
Confounding		Not recorded
Bias of statistics or presentation of result		

**First Author (Year)**	**de Groot B (2017) [23]**
Title	The most commonly used disease severity scores are inappropriate for risk stratification of older emergency department sepsis patients: An observational multi-centre study.
Journal	Scandinavian Journal of Trauma, Resuscitation & Emergency Medicine
Reviewer	RL, CG, MB
Study sponsor	Nil
Study type	Prospectively Collected Data Retrospective Cohort (April 2011–February 2016)
Location	Holland
Participants Number Male/Female Median age Patient group	2280 57.7% male (mean 61.1 years old (SD17.0)) ED patients with suspected infection and Manchester triage category of yellow, orange, or red with IV ABx
qSOFA criteria	Respiratory rate ≥22 bpm, systolic blood pressure ≤100 mmHg, and Glasgow Coma Score (GCS) <15
Primary outcome Other outcomes	In-hospital mortality ICU or MCU admission, an unanticipated transfer to an ICU or MCU within 48 h after being admitted to a ward [20], and the composite outcome of in-hospital mortality, ICU or MCU admission, or unanticipated transfer to an ICU or MCU within 48 h.
Results	AUC (in-hospital mortality?) 0.68 for qSOFA ≥2
Note	17 years old or olderSuspected infection not defined

**Risk of Bias**	**Author’s Judgment** **Low Risk** **Unclear** **High Risk**	**Support for Judgment**
Selection bias		17 or more years old; categories 1–3 only
Bias in definition and measurement		Suspected infection not defined; definition of severe/moderate of severity scores
Outcome measurement bias		
Handling of missing data		
Confounding		
Bias of statistics or presentation of result		

**First Author (Year)**	**Donnelly JP (2017) [24]**
Title	Application of the Third International Consensus Definitions for Sepsis (Sepsis-3) Classification: A retrospective population-based cohort study
Journal	Lancet Infectious Disease
Reviewer	RL, KH, LL, MB, CG
Study sponsor	National Institute of Nursing Research; Center for Clinical and Translational Science and University of Alabama
Study type	Retrospective cohort (January 2003–October 2007)
Location	USA
Participants Number Male/Female Median age Patient group	22692 identified, 2593 analyzed 47.8% male 68 (61–75) Stroke study database; >45 years old; serious infection (defined as requiring admission), All patients (ICU, floor, or others)
qSOFA criteria	Altered mentation (Glasgow coma score <14 or deemed as non-alert on the alert, voice, pain, unresponsive scale), a systolic blood pressure of 100 mm Hg or lower, or respiratory rate of at least 22 breaths per min
Primary outcome Other outcomes	In-hospital mortality 28-day mortality and 1-year mortality
Results	0.759 AUC in-hospital mortality (Baseline plus qSOFA)
Note	

**Risk of Bias**	**Author’s Judgment** **Low Risk** **Unclear** **High Risk**	**Support for Judgment**
Selection bias		Patients from a stroke database, higher African–American population
Bias in definition and measurement		
Outcome measurement bias		
Handling of missing data		
Confounding		
Bias of statistics or presentation of result		

**First Author (Year)**	**Du X (2017) [25]**
Title	Both qSOFA score and bedside plasma lactate are the predictors of mortality for patients with infections in ED.
Journal	American Journal of Emergency Medicine
Reviewer	RL, CG, MB
Study sponsor	Research Fund of the Ministration of Health of China (201302003) and the Ministration of Health of Chengdu City (CDWSYJ-2016-01).
Study type	Retrospective case-controlled study (August 2015–July 2016)
Location	China
Participants Number Male/Female Median age Patient group	565 65.66% male (Mean 56.44 ± 18.1) All ED patients with infections
qSOFA criteria	Respiratory rate ≥22 bpm, systolic blood pressure ≤100 mmHg, and Glasgow Coma Score (GCS) <15
Primary outcome Other outcomes	28-day mortality or/and ICU admission
Results	The odds ratio of qSOFA and plasma lactate were 1.652 and 1.444(p value <0.05)
Note	Correspondence. Short report. Not enough details for study to be analyzed critically

**Risk of Bias**	**Author’s Judgment** **Low Risk** **Unclear** **High Risk**	**Support for Judgment**
Selection bias		
Bias in definition and measurement		Infection not defined
Outcome measurement bias		
Handling of missing data		Large percentage of data missing
Confounding		Unclear, cannot be assessed
Bias of statistics or presentation of result		Unclear, cannot be assessed

**First Author (Year)**	**Finkelsztein EJ (2017) [26]**
Title	Comparison of qSOFA and SIRS for predicting adverse outcomes of patients with suspicion of sepsis outside the intensive care unit
Journal	Critical Care
Reviewer	RL, KH, LL, MB, CG
Study sponsor	National Institutes of Health Grants
Study type	Prospectively Collected Data Retrospective Cohort (October 14—?)
Location	NY, USA
Participants Number Male/Female Median age (95% CI) Patient group	186 identified, 152 analyzed 31% male 64 (51–75) ED or ward to ICU, suspicion of infection
qSOFA criteria	Systolic blood pressure of ≤100 mmHg, respiratory rate of ≥22/minute, and altered mental status. The latter was not confined to a Glasgow Coma Scale score of <15, but it included any altered mentation, such as disorientation and somnolence
Primary outcome Other outcomes	All-cause in-hospital mortality ICU-free days from ICU admission to day 28, ventilator-free days from initiation of invasive mechanical ventilation to day 28, organ dysfunction-free days and renal dysfunction free days from ICU admission to day 14
Results	In-hospital AUC 0.74 (0.66–0.81), Sensitivity 90% (73–98), Specificity 42% (33–52)
Note	

**Risk of Bias**	**Author’s Judgment** **Low Risk** **Unclear** **High Risk**	**Support for Judgment**
Selection bias		Biobank registry. Gender differences were high
Bias in definition and measurement		Individual biases
Outcome measurement bias		
Handling of missing data		Not reported
Confounding		High numbers of malignancy and immunosuppression
Bias of statistics or presentation of result		

**First Author (Year)**	**Forward E (2017) [27]**
Title	Predictive validity of the qSOFA criteria for sepsis in non-ICU inpatients.
Journal	Intensive Care Medicine
Reviewer	RL, CG, MB
Study sponsor	Nil
Study type	Prospective case-controlled study (May–August 15)
Location	Sydney, Australia
Participants Number Male/Female Median age Patient group	161 55% male (mean 70 years old) Adult non-ICU inpatients who triggered the hospital ‘Sepsis Kills’ pathway with acute deterioration and suspected or proven infection
qSOFA criteria	respiratory rate ≥22 bpm, systolic blood pressure ≤100 mmHg, and ‘altered mentation’
Primary outcome Other outcomes	Inpatient sepsis, in-hospital mortality, ICU admission, and blood culture positivity
Results	?
Note	

**Risk of Bias**	**Author’s Judgment** **Low Risk** **Unclear** **High Risk**	**Support for Judgment**
Selection bias		Triggering of pathway
Bias in definition and measurement		Prone to human error
Outcome measurement bias		Cannot be assessed
Handling of missing data		12% missing with no accounting system
Confounding		Cannot be assessed
Bias of statistics or presentation of result		Error in Table 1

**First Author (Year)**	**Freund Y (2017) [28]**
Title	Prognostic accuracy of sepsis-3 criteria for in-hospital mortality among patients with suspected infection presenting to the emergency department
Journal	JAMA
Reviewer	RL, KH, LL, MB, CG
Study sponsor	French Society of Emergency Medicine
Study type	Prospective cohort (16 May 16–16 June)
Location	International: France, Switzerland, Spain, Belgium
Participants Number Male/Female Median age (IQR) Patient group	1088 identified, 879 analyzed 53% male 67 (48–81) ED patients with clinical suspicion of infection
qSOFA criteria	Respiratory rate >21 breaths/min; Systolic arterial blood pressure ≤100 mm Hg; or altered mental status (determined clinically by the treating physician)
Primary outcome Other outcomes	In-hospital mortality Admission to ICU, length of ICU stay of more than 72 h, a composite of death, or ICU stay of more than 72 h
Results	In-hospital mortality AUC 0.80 (0.74–0.85) Sensitivity 70% (59–80), Specificity 79% (76–82), PPV 24% (18–30), NPV 97% (95–98)
Note	

**Risk of Bias**	**Author’s Judgment** **Low Risk** **Unclear** **High Risk**	**Support for Judgment**
Selection bias		
Bias in definition and measurement		Altered mental status (determined clinically by the treating physician)
Outcome measurement bias		
Handling of missing data		
Confounding		
Bias of statistics or presentation of result		

**First Author (Year)**	**Giamarellos-Bournoulis EJ (2017) [29]**
Title	Validation of the new Sepsis-3 definitions: Proposal for improvement in early risk identification
Journal	Clinical Microbiology and Infection
Reviewer	RL, KH, LL, MB, CG
Study sponsor	Hellenic Institute for the Study of Sepsis
Study type	Retrospective cohort (May 06–Decemeber 15)
Location	Greece
Participants Number Male/Female Median age Patient group	5176 identified, 4487 analyzed ? 76 (22) All patients with signs of infection of onset <24 h ago and at least two signs of SIRS
qSOFA criteria	GCS <13, RR>22, sBP <100
Primary outcome Other outcomes	Sensitivity of qSOFA and of the new sepsis definition to predict 28-day mortality To compare the performance of qSOFA and SIRS criteria for the early prediction of organ dysfunction outside the ICU, and to compare misclassification of severe cases by the 1991 definitions, and by Sepsis-3 definitions separately for non-ICU and ICU patients
Results	?
Note	

**Risk of Bias**	**Author’s Judgment** **Low Risk** **Unclear** **High Risk**	**Support for Judgment**
Selection bias		High threshold for inclusion criteria
Bias in definition and measurement		High threshold for altered mentation, respiratory rate, and systolic blood pressure
Outcome measurement bias		Not defined clearly
Handling of missing data		Not stated
Confounding		No population characteristics and co-morbidities
Bias of statistics or presentation of result		Too limited to be commented on

**First Author (Year)**	**González del Castillo (2017) [30]**
Title	Prognostic accuracy of SIRS criteria, qSOFA score and GYM score for 30-day-mortality in older non-severely dependent infected patients attended in the emergency department.
Journal	European Journal of Clinical Microbiology & Infectious Diseases
Reviewer	RL, CG, KH
Study sponsor	No financial support was used. The promoter of this study has been the Infectious Disease Group of the Spanish Emergency Medicine Society. This group has received financial support from Merck, Tedec-Meiji, Pfizer, Thermo Fisher, Laboratorios Rubio and Novartis in the last year to organize conferences and group meetings. None of the authors have received any financial compensation.
Study type	Observational, prospective cohort study (1 and 22 October 2015, 12 and 19 January 2016, and 13 and 27 April 2016)
Location	Spain
Participants Number Male/Female Median age Patient group	1071 50.8% male (mean 83.6 (SD 5.6)) Patients aged 75 years or older who attended for an acute infection, who did not have severe functional dependence (Barthel index >40)
qSOFA criteria	Glasgow Coma Scale score <15, systolic blood pressure < 100 mmHg and respiratory rate ≥ 22 per min
Primary outcome Other outcomes	All-cause 30-day mortality
Results	All-cause 30-day mortality AUC 0.69 (95% CI 0.61–0.76) for the qSOFA score
Note	

**Risk of Bias**	**Author’s Judgment** **Low Risk** **Unclear** **High Risk**	**Support for Judgment**
Selection bias		Older patients. Barthel index >40
Bias in definition and measurement		SIRS definition, GCS defined differently
Outcome measurement bias		
Handling of missing data		Not reported
Confounding		
Bias of statistics or presentation of result		

**First Author (Year)**	**Guirgis (2017) [31]**
Title	Development of a Simple Sequential Organ Failure Assessment Score for Risk Assessment of Emergency Department Patients with Sepsis
Journal	Journal of Intensive Care Medicine
Reviewer	RL, CG, KH
Study sponsor	National Institutes of General Medical Sciences and NIH Loan Repayment Program
Study type	Retrospective cohort (October 13–May 16)
Location	Jacksonville, FL, USA
Participants Number Male/Female Median age Patient group	3297 49% male 59 (48–70) Adult patients admitted through ED and discharge diagnosis of sepsis
qSOFA criteria	respiratory rate ≥22 breaths/ minute, altered mental status, or systolic blood pressure ≤100 mm Hg
Primary outcome Other outcomes	in-hospital mortality Sensitivities and specificities were calculated for patients with a discharge diagnosis of sepsis with a score of 2 or more for SOFA, qSOFA, or simple SOFA and were compared to patients with a score of <2
Results	In-hospital mortality AUC 0.68 for qSOFA sensitivity and specificity of qSOFA ≥2 were 38% and 86%, respectively
Note	

**Risk of Bias**	**Author’s Judgment** **Low Risk** **Unclear** **High Risk**	**Support for Judgment**
Selection bias		
Bias in definition and measurement		AMS relied on nursing documentation
Outcome measurement bias		
Handling of missing data		Listed as missing but not accounted for
Confounding		
Bias of statistics or presentation of result		

**First Author (Year)**	**Haydar S (2017) [32]**
Title	Comparison of QSOFA score and SIRS criteria as screening mechanisms for emergency department sepsis.
Journal	American Journal of Emergency Medicine
Reviewer	RL, CG, KH
Study sponsor	Nil
Study type	Retrospective study (September 14–September 15)
Location	USA
Participants Number Male/Female Median age Patient group	199 55% male 71 years old (range 18–102) Adult septic Medicare and Medicaid patients treated with antibiotics in the ED for suspected infection, admitted to the hospital, and subsequently discharged with a Center for Medicare Services Diagnosis Related Grouping (DRG) for sepsis
qSOFA criteria	Altered mental status (AMS), respiratory rate (RR) >22/min, and systolic blood pressure (SBP) <100 mmHg
Primary outcome Other outcomes	Sensitivity of the qSOFA score in diagnosing sepsis Diagnostic timeliness of qSOFA in diagnosing sepsis when compared to the traditional SIRS criteria
Results	AUC 0.68 (0.58–0.78) for qSOFA
Note	

**Risk of Bias**	**Author’s Judgment** **Low Risk** **Unclear** **High Risk**	**Support for Judgment**
Selection bias		Medicare and Medicaid patients only
Bias in definition and measurement		AMS, diagnosis, and suspected infection not defined
Outcome measurement bias		
Handling of missing data		Not accounted for
Confounding		
Bias of statistics or presentation of result		

**First Author (Year)**	**Henning DJ [33]**
Title	An Emergency Department Validation of the SEP-3 Sepsis and Septic Shock Definitions and Comparison With 1992 Consensus Definitions
Journal	Annals of Emergency Medicine
Reviewer	RL, KH, LL, MB, CG
Study sponsor	Non stated
Study type	Prospectively Collected Data Retrospective Cohort (3 Decemeber–4 September, 5 September –6 September, 4 July–5 June)
Location	USA
Participants Number Male/Female Median age (SD) Patient group	7637 identified, 7754 analyzed 52.2% male 56.9 (20.8) All patients (ED, ward, ICU) with suspected infection
qSOFA criteria	Respiratory rate greater than or equal to 22 breaths/min, altered mental status (documented by physician), and hypotension defined by a systolic blood pressure of less than or equal to 100 mm Hg.
Primary outcome Other outcomes	All-cause in-hospital mortality, defined as death before hospital discharge. -
Results	AUC 0.77, Sens 52(46–57), Spec 86(85–87), PPV 14(13–15), NPV 98(98–98)
Note	

**Risk of Bias**	**Author’s Judgment** **Low Risk** **Unclear** **High Risk**	**Support for Judgment**
Selection bias		
Bias in definition and measurement		Subject to individual bias
Outcome measurement bias		
Handling of missing data		
Confounding		
Bias of statistics or presentation of result		

**First Author (Year)**	**Ho KM (2017) [34]**
Title	Combining quick Sequential Organ Failure Assessment with plasma lactate concentration is comparable to standard Sequential Organ Failure Assessment score in predicting mortality of patients
Journal	Journal of Critical Care
Reviewer	RL, KH, LL, MB, CG
Study sponsor	Department of Intensive Care Medicine, Royal Perth Hospital
Study type	Prospectively Collected Data Retrospective Cohort (8 January–13 Decemeber)
Location	Australia
Participants Number Male/Female Median Age (IQR) Patient group	9549 identified, 2322 analyzed 61% male 57.1 (41–70) All ICU patient during the first hour of admission
qSOFA criteria	Respiration rate ≥22 breaths/min, altered mental state (Glasgow Coma Scale score <15), and systolic blood pressure ≤100 mm Hg
Primary outcome Other outcomes	(In)hospital mortality Patients who required invasive mechanical ventilation within 24 h of ICU admission, and a length of ICU stay more than 10 days
Results	In-hospital mortality AUC 0.672 (0.638–0.707)
Note	

**Risk of Bias**	**Author’s Judgment** **Low Risk** **Unclear** **High Risk**	**Support for Judgment**
Selection bias		Database included ICU patients only, Gender imbalance
Bias in definition and measurement		
Outcome measurement bias		
Handling of missing data		
Confounding		Identified but not adjusted for
Bias of statistics or presentation of result		

**First Author (Year)**	**Hwang SY (2018) [36]**
Title	Low Accuracy of Positive qSOFA Criteria for Predicting 28-Day Mortality in Critically Ill Septic Patients During the Early Period After Emergency Department Presentation.
Journal	Annals of Emergency Medicine
Reviewer	RL, CG, KH
Study sponsor	Nil
Study type	Retrospective cohort study (August 08–September 14)
Location	Seoul, S Korea
Participants Number Male/Female Median age Patient group	1395 56% male 65 (55–73) Patients aged 18 years or older and who received a diagnosis of severe sepsis or septic shock (defined by SIRS) during their ED stay were included in analysis
qSOFA criteria	Systolic blood pressure of less than or equal to 100 mmHg, respiratory rate greater than or equal to 22 breaths/min, and altered mentation (GCS < 15 or <Alert on AVPU)
Primary outcome Other outcomes	28-day mortality In-hospital mortality, use of a vasopressor within 24 h after ED presentation, presence of cryptic shock, increase in a SOFA score of 2 points or more from the baseline, ICU admission, and mechanical ventilation
Results	28-day mortality AUC 0.58 (95% CI 0.55 to 0.62) on ED arrival for qSOFA ≥2
Note	Neutropenic patients included

**Risk of Bias**	**Author’s Judgment** **Low Risk** **Unclear** **High Risk**	**Support for Judgment**
Selection bias		Severe sepsis/septic shock. Patients not for active treatments were excluded.
Bias in definition and measurement		
Outcome measurement bias		
Handling of missing data		Missing cases excluded
Confounding		
Bias of statistics or presentation of result		

**First Author (Year)**	**Innocenti F (2018) [37]**
Title	SOFA score in septic patients: Incremental prognostic value over age, comorbidities, and parameters of sepsis severity.
Journal	Internal & Emergency Medicine
Reviewer	RL, CG, LL
Study sponsor	Nil
Study type	Retrospective review (June 08–April 16)
Location	ED-HDU
Participants Number Male/Female Median age Patient group	742 53% male (mean age 75 ± 14) Diagnosis of sepsis, severe sepsis, or septic shock.
qSOFA criteria	GCS < 15 or AVPU, others were not defined
Primary outcome Other outcomes	28-day mortality ICU admission
Results	qSOFA 0.625, 95%, CI 0.579–0.671
Note	

**Risk of Bias**	**Author’s Judgment** **Low Risk** **Unclear** **High Risk**	**Support for Judgment**
Selection bias		Change of definition through time.Sick population. ED HDU patient
Bias in definition and measurement		AMS—determined by deduction from notesSepsis was defined by the 2001 definition
Outcome measurement bias		
Handling of missing data		
Confounding		31% mortality
Bias of statistics or presentation of result		Statistics unclearDouble-counting MEWS and SOFA in modelling

**First Author (Year)**	**Khwannimit B (2017) [38]**
Title	Comparison of the performance of SOFA, qSOFA and SIRS for predicting mortality and organ failure among sepsis patients admitted to the intensive care unit in a middle-income country.
Journal	Journal of Critical Care
Reviewer	RL, CG, KH
Study sponsor	Research grant of Faculty of Medicine, Prince of Songkla University
Study type	Retrospective cohort study (07–16)
Location	Thailand
Participants Number Male/Female Median age Patient group	2350 56.1% male 62 (45–75) 15 years and older who had been diagnosed with sepsis and admitted to a medical intensive care unit (sepsis was defined by the criteria of the international consensus definition of sepsis) Definitions Conference (Sepsis-2)
qSOFA criteria	SBP ≤100 mmHg, respiratory rate ≥22 breath/min, and Glasgow Coma Score (GCS) ≤13
Primary outcome Other outcomes	All-cause hospital mortality ICU mortality and organ failure
Results	All-cause hospital mortality AUC 0.814 for qSOFA
Note	

**Risk of Bias**	**Author’s Judgment** **Low Risk** **Unclear** **High Risk**	**Support for Judgment**
Selection bias		MICU patients, 15+ years old
Bias in definition and measurement		Sepsis 2 definition of sepsis
Outcome measurement bias		
Handling of missing data		
Confounding		
Bias of statistics or presentation of result		

**First Author (Year)**	**Kim MW (2017) [39]**
Title	Mortality prediction using serum biomarkers and various clinical risk scales in community-acquired pneumonia.
Journal	Scandinavian Journal of Clinical & Laboratory Investigation
Reviewer	RL, CG, KH
Study sponsor	Nil
Study type	Retrospective chart review (January–Decemeber 14)
Location	Seoul Korea
Participants Number Male/Female Median age Patient group	125 62.4% male 72 years (59.5–80.0) In-patient adults with a diagnosis of Community Acquired Pneumonia (CAP)
qSOFA criteria	Respiratory rate of 22/min or greater, altered mentation (AVPU), or systolic blood pressure of 100 mmHg or less
Primary outcome Other outcomes	Evaluate the performance of various biomarkers and other clinical risk scales for predicting 28-day mortality in CAP patients who were admitted to the ED, and to compare the performance of these predictors.
Results	28-day mortality AUC 0.81 for qSOFA ≥2
Note	

**Risk of Bias**	**Author’s Judgment** **Low Risk** **Unclear** **High Risk**	**Support for Judgment**
Selection bias		CAP
Bias in definition and measurement		
Outcome measurement bias		
Handling of missing data		Not identified or addressed
Confounding		CAP patients
Bias of statistics or presentation of result		Significant amounts of missing data

**First Author (Year)**	**Kolditz M (2016) [40]**
Title	Comparison of the qSOFA and CRB-65 for risk prediction in patients with community-acquired pneumonia
Journal	Intensive Care Medicine
Reviewer	RL, KH, LL, MB, CG
Study sponsor	CAPNETZ was founded by a BMBF Grant (01KI07145) 2001–2011.
Study type	Retrospective cohort (Letter) (2 October–15 June)
Location	Germany
Participants Number Male/Female Median age (IQR) Patient group	9327 analyzed 56% male 63 ICU patients included in a German community-acquired pneumonia database
qSOFA criteria	Respiratory rate ≥22/min, systolic blood pressure ≤100 mmHg, pneumonia-related (new-onset) confusion according to the physician’s discretion
Primary outcome Other outcomes	30-day mortality Requirement for mechanical ventilation and/or vasopressor support during hospital admission, and the combination of 30-day mortality and requirement for mechanical ventilation and/or vasopressor
Results	In-hospital mortality AUC 0.70 (0.69–0.71)
Note	

**Risk of Bias**	**Author’s Judgment** **Low Risk** **Unclear** **High Risk**	**Support for Judgment**
Selection bias		Pneumonia database, inclusion bias
Bias in definition and measurement		Subject to individual bias
Outcome measurement bias		
Handling of missing data		Missing data excluded from database
Confounding		None found
Bias of statistics or presentation of result		

**First Author (Year)**	**LeGuen M (2018) [41]**
Title	Frequency and significance of qSOFA criteria during adult rapid response team reviews: A prospective cohort study.
Journal	Resuscitation
Reviewer	RL, CG, KH
Study sponsor	Nil
Study type	prospective observational audit 6 June, 10 July 16
Location	Victoria, Australia
Participants Number Male/Female Median age Patient group	258 48% male 72 (57–82) Adults requiring Rapid Response Team response
qSOFA criteria	Altered mentation (as measured by a GCS <15); Respiratory Rate ≥22/min; SBP ≤100 mmHg
Primary outcome Other outcomes	In-hospital mortality as per the original qSOFA study ICU length of stay more than three days [6], death, or ICU length of stay greater than three days, intensity of ICU supports, and discharge destination.
Results	
Note	

**Risk of Bias**	**Author’s Judgment** **Low Risk** **Unclear** **High Risk**	**Support for Judgment**
Selection bias		
Bias in definition and measurement		
Outcome measurement bias		
Handling of missing data		10% excluded
Confounding		
Bias of statistics or presentation of result		Easily misinterpreted

**First Author (Year)**	**Moskowitz A (2017) [42]**
Title	Quick Sequential Organ Failure Assessment and Systemic Inflammatory Response Syndrome Criteria as Predictors of Critical Care Intervention Among Patients With Suspected Infection.
Journal	Critical Care Medicine
Reviewer	RL, CG, MB
Study sponsor	Drs. Moskowitz, Chase, Berg, and Donnino received support for the article research from the National Institutes of Health (NIH). Dr. Moskowitz is funded by a grant from the NIH (2T32HL007374-37). Dr. Chase is funded by a grant from the National Institute of General Medical Sciences (K23 GM101463). Dr. Shapiro received funding from Thermo Fisher, Cheetah Medical, Rapid Pathogen Screening, and Baxter. Dr. Cocchi is funded by a grant from the American Heart Association (15SDG22420010). Dr. Berg is funded by a grant from the National Institute of Heart, Lung and Blood Institute (NIHLBI) (K23HL128814-01A1). Dr. Donnino is funded by a grant from the NIHLBI (1K24HL127101).
Study type	Retrospective cohort
Location	United States (January 2010 and December 2014)
Participants Number Male/Female Median age Patient group	24,164 50.9% male (Mean 63.8 (SD 18.1)) Patients admitted to ED with suspected infection (defined by the collection of any microbial cultures and initiation of antibiotics within 24 h of ED triage time
qSOFA criteria	Not defined
Primary outcome Other outcomes	“Received CCI” within 48 h of ED triage Nil
Results	AUC 0.71 (0.69–0.72) when used to predict the in-hospital mortality

**Risk of Bias**	**Author’s Judgment** **Low Risk** **Unclear** **High Risk**	**Support for Judgment**
Selection bias		
Bias in definition and measurement		Unclear definition
Outcome measurement bias		Not objective
Handling of missing data		
Confounding		
Bias of statistics or presentation of result		

**First Author (Year)**	**Muller M (2017) [43]**
Title	Utility of quick sepsis-related organ failure assessment (qSOFA) to predict outcome in patients with pneumonia.
Journal	PLoS ONE
Reviewer	RL, CG, MB
Study sponsor	Nil
Study type	Retrospective analysis (June 11–May 13)
Location	Switzerland
Participants Number Male/Female Median age Patient group	527 64.5% male 66 (50–76) Adults (16 years or older) presenting with a diagnosis of pneumonia
qSOFA criteria	Glasgow Coma Scale (GCS) of 14 or less, systolic blood pressure of 100 mmHg or less, respiration rate of 22/min or more.
Primary outcome Other outcomes	In-hospital mortality ICU admission rate and length of hospital stay
Results	In-hospital mortality AUC 0.58 for qSOFA
Note	

**Risk of Bias**	**Author’s Judgment** **Low Risk** **Unclear** **High Risk**	**Support for Judgment**
Selection bias		Pneumonia only
Bias in definition and measurement		
Outcome measurement bias		
Handling of missing data		Patients excluded but not explained
Confounding		
Bias of statistics or presentation of result		Presentation of wrong results from calculations

**First Author (Year)**	**Park HK (2017) [44]**
Title	Quick sequential organ failure assessment compared to systemic inflammatory response syndrome for predicting sepsis in emergency department.
Journal	Journal of Critical Care
Reviewer	RL, CG, MB
Study sponsor	Nil
Study type	Retrospective cohort March 07–February 16
Location	Seoul Korea
Participants Number Male/Female Median age Patient group	1009 45% male (Mean 67.4 ± 17.6) Patients (≥18 years) with a suspected infection that was identified by using a combination of antibiotics (oral or parenteral) and body fluid cultures (blood, urine, cerebrospinal fluid, etc.)
qSOFA criteria	respiratory rate ≥22/min, systolic blood pressure ≤100 mm Hg, and altered mentation (all cases except ‘alert’ were judged to have altered mentation)
Primary outcome Other outcomes	Increase of 2 or more SOFA points within 24 h of ED admission In-hospital mortality
Results	In-hospital mortality AUC 0.733 for qSOFA
Note	

**Risk of Bias**	**Author’s Judgment** **Low Risk** **Unclear** **High Risk**	**Support for Judgment**
Selection bias		
Bias in definition and measurement		Retrospective with antibiotic cultures only
Outcome measurement bias		
Handling of missing data		Identified but not addressed
Confounding		Retrospective study, time bias
Bias of statistics or presentation of result		Calibration unclear

**First Author (Year)**	**Peake (2017) [45]**
Title	Potential Impact of the 2016 Consensus Definitions of Sepsis and Septic Shock on Future Sepsis Research.
Journal	Annals of Emergency Medicine
Reviewer	RL, CG, LL
Study sponsor	Nil
Study type	Post hoc analysis of ARISE database (October 08–April 14)
Location	Australasia
Participants Number Male/Female Median age Patient group	1591 59.7/40.3 (Mean 62.9, SD 16.5) SIRS-positive adults
qSOFA criteria	≥22 breaths/min, systolic blood pressure ≤100 mm Hg, Glasgow Coma Scale [GCS] score <15
Primary outcome Other outcomes	The proportion of patients enrolled with the SIRS-based criteria that met the new Sepsis-3 definitions for qSOFA, sepsis, and septic shock their baseline characteristics; interventions delivered; and outcomes, including mortality, duration of organ support, and ICU, and the hospital length of stay
Results	
Note	Second analysis of ARISE database Multiple imputation for Sn, Sp, PPV, and NPV

**Risk of Bias**	**Author’s Judgment** **Low Risk** **Unclear** **High Risk**	**Support for Judgment**
Selection bias		Retrospective data that included patients with SIRS-based criteria only
Bias in definition and measurement		
Outcome measurement bias		
Handling of missing data		Unclear
Confounding		
Bias of statistics or presentation of result		

**First Author (Year)**	**Quinten VM (2017) [46]**
Title	Sepsis patients in the emergency department—Stratification using the Clinical Impression Score, Predisposition, Infection, Response and Organ dysfunction score
Journal	European Journal of Emergency Medicine
Reviewer	RL, KH, LL,
Study sponsor	Not stated
Study type	Prospectively Collected Data Retrospective Cohort (August 12–April 14)
Location	Netherlands
Participants Number Male/Female Mean age (IQR) Patient group	193 analyzed 56% male 60 (48–71) Non-traumatic patients with suspected infection or sepsis in the ED
qSOFA criteria	Altered mental status, respiratory frequency, and systolic blood pressure.
Primary outcome Other outcomes	ICU admission In-hospital, 28-day and 6-month mortality, indirect admission to the ICU, and length of stay
Results	In-hospital mortality AUC 0.823 (0.707–0.939)
Note	

**Risk of Bias**	**Author’s Judgment** **Low Risk** **Unclear** **High Risk**	**Support for Judgment**
Selection bias		
Bias in definition and measurement		Not defined
Outcome measurement bias		Subject to individual bias
Handling of missing data		
Confounding		
Bias of statistics or presentation of result		Number of missing data (that was excluded) is not stated

**First Author (Year)**	**Raith EP (2017) [47]**
Title	Prognostic accuracy of the SOFA score, SIRS criteria, and qSOFA score for in-hospital mortality among adults with suspected infection admitted to the intensive care unit
Journal	JAMA
Reviewer	RL, KH, LL,
Study sponsor	Competitive Research Financing of Tampere University Hospital
Study type	Retrospective cohort (2000–2015)
Location	Australasia
Participants Number Male/Female Mean age (SD) Patient group	1,499,753 identified, 184,875 analyzed 55.4% male 62.9 (17.4) ICU patients with infection-related diagnosis
qSOFA criteria	A Glasgow Coma Scale of less than 15 (others not stated)
Primary outcome Other outcomes	In-hospital mortality Combination of in-hospital mortality, or an ICU length of stay of three days or longer
Result	In-hospital mortality AUC 0.607 (99% CI 0.603–0.611)
Note	

**Risk of Bias**	**Author’s Judgment** **Low Risk** **Unclear** **High Risk**	**Support for Judgment**
Selection bias		
Bias in definition and measurement		
Outcome measurement bias		
Handling of missing data		
Confounding		
Bias of statistics or presentation of result		

**First Author (Year)**	**Rannikko J (2017) [48]**
Title	Sepsis-related mortality in 497 cases with blood culture-positive sepsis in an emergency department
Journal	International Journal of Infectious Diseases
Reviewer	RL, KH, LL,
Study sponsor	Competitive Research Financing of Tampere University Hospital
Study type	Retrospective cohort (March 12–February 14)
Location	Finland
Participants Number Male/Female Median Age (IQR) Patient group	800 identified, 497 analyzed 53% male 68 (58–78) ED patients with positive blood culture results
qSOFA criteria	Respiratory rate > 22/min, altered mentation (GCS < 15), and systolic blood pressure < 100 mmHg
Primary outcome Other outcomes	90-day mortality 28-day mortality
Results	Patients with missing data and under 18 years old are excluded, sample size 473. 28-day mortality AUC 0.71 (0.67–0.75), Sensitivity 0.65 (0.53–0.76), Specificity 0.77 (0.73–0.81), PPV 0.33 (0.28–0.39), NPV 0.93(0.9–0.95) +LR 2.9 (2.26–3.72), −LR 0.45 (0.32–0.62)
Note	

**Risk of Bias**	**Author’s Judgment** **Low Risk** **Unclear** **High Risk**	**Support for Judgment**
Selection bias		Blood culture-positive only
Bias in definition and measurement		Altered mentation not defined in the original article, contacted author for clarification
Outcome measurement bias		
Handling of missing data		
Confounding		
Bias of statistics or presentation of result		Limited statistics in the original paper. However the original author has supplied our team with de-personalized raw data for further data analysis

**First Author (Year)**	**Ranzani (2017) [49]**
Title	New Sepsis Definition (Sepsis-3) And Community-Acquired Pneumonia Mortality—A Validation and Clinical Decision-Making Study
Journal	American Journal of Respiratory and Critical Care Medicine
Reviewer	RL, CG, LL
Study sponsor	Centro de Investigacio’ n Biomedica En Red-Enfermedades Respiratorias and the European Respiratory Society Research Fellowships
Study type	Prospectively Collected Data Retrospective Cohort (1996–2015)
Location	Barcelona and Valencia
Participants Number Male/Female Mean Age (SD) Patient group	6874 62.2 Male Mean (66.1 (19)) Clinical diagnosis of CAP
qSOFA criteria	≥22 breaths/min, systolic blood pressure ≤100 mm Hg, altered mental status
Primary outcome Other outcomes	In-hospital mortality In-hospital mortality and/or need for critical support for three or more days, and 30-day mortality
Result	In-hospital mortality AUC 0.697 (0.671–0.722) qSOFA >2 Sn 50(45–55), Sp 81 (80–82), PPV 15 (13–17), NPV 96 (96–97), LR+ 2.70 (2.41–3.03), LR- 0.61 (0.55–0.68)
Note	

**Risk of Bias**	**Author’s Judgment** **Low Risk** **Unclear** **High Risk**	**Support for Judgment**
Selection bias		CAP patients. Time bias
Bias in definition and measurement		Confusion not clearly defined
Outcome measurement bias		
Handling of missing data		
Confounding		Secondary analysis, time
Bias of statistics or presentation of result		

**First Author (Year)**	**Seymour CW (2016) [50]**
Title	Assessment of clinical criteria for sepsis for the third international consensus definitions for sepsis and septic shock (Sepsis-3)
Journal	JAMA
Reviewer	RL, KH, LL,
Study sponsor	National Institutes of Health, the Department of Veterans, the Permanente Medical Group, German Federal Ministry of Education and Research
Study type	Retrospective cohort (January 10–Decemeber 12)
Location	US and Germany
Participants Number Male/Female Mean Age (SD) Patient group	1,309,025 identified, 74,453 analyzed 43% male 61 (19) All patients with suspected infection
qSOFA criteria	Systolic hypotension (<100 mmHg), tachypnea (>22/min), or altered mentation GCS < 13
Primary outcome Other outcomes	In-hospital mortality Combination of in-hospital mortality or ICU stay
Result	
Note	

**Risk of Bias**	**Author’s Judgment** **Low Risk** **Unclear** **High Risk**	**Support for Judgment**
Selection bias		Multiple databases used. Potential bias in individual database
Bias in definition and measurement		Altered mentation not defined
Outcome measurement bias		
Handling of missing data		
Confounding		
Bias of statistics or presentation of result		

**First Author (Year)**	**Siddiqui S (2017) [51]**
Title	A comparison of pre ICU admission SIRS, EWS and qSOFA scores for predicting mortality and length of stay in ICU
Journal	Journal of Critical Care
Reviewer	RL, CG, MB
Study sponsor	Nil
Study type	Retrospective cohort (January–Decemeber 15)
Location	Singapore
Participants Number Male/Female Median age Patient group	58 60% male (Mean 64.4 ± 12.9) All adult ICU or HDU admissions with a presumed diagnosis of ‘sepsis’
qSOFA criteria	Hypotension b 100 SBP, altered consciousness, GCS b 15, and a respiratory rate N 22 bpm
Primary outcome Other outcomes	In-hospital mortality and ICU length of stay Nil
Results	Mortality AUC 0.6875 for qSOFA
Note	

**Risk of Bias**	**Author’s Judgment** **Low Risk** **Unclear** **High Risk**	**Support for Judgment**
Selection bias		Sepsis not defined and unclear
Bias in definition and measurement		Sepsis not defined and unclear
Outcome measurement bias		
Handling of missing data		Not stated. Small number
Confounding		Not enough information for assessment
Bias of statistics or presentation of result		Small number

**First Author (Year)**	**Singer AJ (2017) [52]**
Title	Quick SOFA Scores Predict Mortality in Adult Emergency Department Patients With and Without Suspected Infection
Journal	Annals of Emergency Medicine
Reviewer	RL, KH, LL,
Study sponsor	Nil
Study type	Retrospective cohort (14 January–15 March)
Location	NY, USA
Participants Number Male/Female Mean age (SD) Patient group	67,475 identified, 22,530 analyzed 47% male 54 (21) All ED patients
qSOFA criteria	Respiratory rate ≥22 breaths/min, systolic blood pressure ≤100 mm Hg, and altered mental status
Primary outcome Other outcomes	In-hospital mortality Hospital admission, ICU admission, and total hospital length of stay (ED triage to discharge from the hospital)
Results	AUC in-hospital mortality 0.76 (95% CI 0.71–0.78), Sen 29% (95% CI 25% to 34%), and spec 97% (95% CI 97% to 97%), respectively, with a NPV of 99% (95% CI 99% to 99%).
Note	

**Risk of Bias**	**Author’s Judgment** **Low Risk** **Unclear** **High Risk**	**Support for Judgment**
Selection bias		
Bias in definition and measurement		Not stated explicitly, presumably the level of consciousness
Outcome measurement bias		
Handling of missing data		Large number (61.3%) of missing data excluded
Confounding		Not stated
Bias of statistics or presentation of result		Not enough to judge

**First Author (Year)**	**Sterling (2017) [53]**
Title	The Impact of the Sepsis-3 Septic Shock Definition on Previously Defined Septic Shock Patients.
Journal	Critical Care Medicine
Reviewer	RL, CG, LL
Study sponsor	Dr. Puskarich received support for article research from the National Institutes of Health (NIH), Dr. Guirgis’ institution received funding from the Society of Critical Care Medicine Vision Grant and from National Center for Advancing Translational Sciences through the University of Florida. Dr. Jones receives support through the National Institutes of General Medical Sciences (R01GM103799-01)
Study type	Secondary analysis of two previously completed clinical trials
Location	Large academic emergency departments in the United States.
Participants Number Male/Female Median age Patient group	470 (mean 60 ± 16.7) Patients with suspected infection, more than or equal to two systemic inflammatory response syndrome criteria, and systolic blood pressure of less than 90 mm Hg after fluid resuscitation.
qSOFA criteria	(respiratory rate ≥ 22 beats/min, altered mental status, or systolic blood pressure (SBP) of ≤ 100 mm Hg)
Primary outcome Other outcomes	In-hospital mortality
Results	
Note	57% of patients meeting old definition for septic shock did not meet Sepsis-3 criteria

**Risk of Bias**	**Author’s Judgment** **Low Risk** **Unclear** **High Risk**	**Support for Judgment**
Selection bias		Patient defined altered mentation. Sick population, inclusion by SIRS
Bias in definition and measurement		Suspected infection and SIRS patients, and sBP less than 90 mmHg
Outcome measurement bias		
Handling of missing data		
Confounding		Secondary analysis
Bias of statistics or presentation of result		

**First Author (Year)**	**Szakmany (2018) [54]**
Title	Defining sepsis on the wards: Results of a multi-centre point-prevalence study comparing two sepsis definitions
Journal	Anaesthesia
Reviewer	RL, CG, MB
Study sponsor	Fiona Elizabeth Agnew Trust and the Welsh Intensive Care Society
Study type	Prospective observational study (19 October 2016)
Location	Wales
Participants Number Male/Female Median age Patient group	380 47% male 74 (61–83) Patients in the ED or in an acute in-patient ward setting with suspected or proven infection
qSOFA criteria	Systolic blood pressure ≤ 100 mmHg, respiratory rate ≥ 22 breaths/min, and altered mental status (defined as either a Glasgow Coma Scale score ≤ 13 or an Alert Voice Pain Unresponsive scale (AVPU) other than ‘Alert’)
Primary outcome Other outcomes	Mortality within 30 days Presence of organ dysfunction defined by SOFA score > 2 or the presence of ‘severe sepsis’
Results	AUC for 30-day mortality 0.57 (0.49–0.64) *p* = 0.07, Sen 0.22 (0.14–0.33), Spec 0.89 (0.85–0.92), PPV 0.34 (0.22–0.49), NPV 0.82 (0.77–0.85)
Note	

**Risk of Bias**	**Author’s Judgment** **Low Risk** **Unclear** **High Risk**	**Support for Judgment**
Selection bias		NEWS of 3 or more
Bias in definition and measurement		Sepsis = qsofa of 2 or more
Outcome measurement bias		
Handling of missing data		No indication on how it is handled
Confounding		Not stated
Bias of statistics or presentation of result		Logistic regression not calibrated

**First Author (Year)**	**Tusgul (2017) [55]**
Title	Low sensitivity of qSOFA, SIRS criteria and sepsis definition to identify infected patients at risk of complication in the prehospital setting and at the emergency department triage
Journal	Scandinavian Journal of Trauma, Resuscitation and Emergency Medicine
Reviewer	RL, CG, LL
Study sponsor	Nil
Study type	Retrospective cohort
Location	Switzerland
Participants Number Male/Female Median age Patient group	886 52.1% male 80 (69–87) Patients transported by an ambulance crew with criteria fulfilling diagnosis or suspicion of infection
qSOFA criteria	SBP ≤100 mmHg, RR ≥22/min, and GCS<15, or altered mental status from baseline as reported by the family
Primary outcome Other outcomes	Predict ICU admission, ICU stay of ≥3 days and mortality at 48 h.
Results	?
Note	Pre-hospital

**Risk of Bias**	**Author’s Judgment** **Low Risk** **Unclear** **High Risk**	**Support for Judgment**
Selection bias		
Bias in definition and measurement		
Outcome measurement bias		
Handling of missing data		Small number, excluded
Confounding		Only one reviewer reviewed the charts
Bias of statistics or presentation of result		

**First Author (Year)**	**Umemura (2017) [56]**
Title	Assessment of mortality by qSOFA in patients with sepsis outside ICU: A post hoc subgroup analysis by the Japanese Association for Acute Medicine Sepsis Registry Study Group.
Journal	Journal of Infection and Chemotherapy
Reviewer	RL, CG, MB
Study sponsor	Nil
Study type	Prospectively Collected Data Retrospective Cohort
Location	Japan
Participants Number Male/Female Median age Patient group	387 59.7% male ? Adults diagnosed with ‘severe sepsis’ as defined in 2003
qSOFA criteria	Altered mental status (Glasgow Coma Scale score of ≤14), systolic blood pressure of less than or equal to 100 mmHg, and a respiratory rate of at least 22 breaths/min
Primary outcome Other outcomes	All-cause in-hospital mortality ?
Results	In-hospital mortality AUC 0.615 for qSOFA
Note	

**Risk of Bias**	**Author’s Judgment** **Low Risk** **Unclear** **High Risk**	**Support for Judgment**
Selection bias		Old definition, “severe sepsis”, time bias
Bias in definition and measurement		
Outcome measurement bias		
Handling of missing data		Not stated, unclear
Confounding		
Bias of statistics or presentation of result		Little to interpret, logistic regression not calibrated

**First Author (Year)**	**Wang, J.Y. (2016) [57]**
Title	Predictive performance of quick Sepsis-related Organ Failure Assessment for mortality and ICU admission in patients with infection at the ED
Journal	American Journal of Emergency Medicine
Reviewer	RL, KH, LL,
Study sponsor	Nil
Study type	Prospectively collected data retrospective cohort (July 15–Decemeber 15)
Location	Beijing, China
Participants Number Male/Female Mean age (SD) Patient group	516 identified, 477 analyzed 61.8%male 73 (60–79) ED patients with a “clinical” diagnosis of infection
qSOFA criteria	Glasgow Coma Scale score of less than or equal to 13, systolic blood pressure less than or equal to 100 mm Hg, and respiratory rate greater than or equal to 22 per minute
Primary outcome Other outcomes	28-day mortality Admission to ICU
Results	28-day mortality AUC 0.666 (95% CI 0.609–0.723), Sen 42.9%, spec 82.6%, PPV 61.8%, NPV 68.8%
Note	

**Risk of Bias**	**Author’s Judgment** **Low Risk** **Unclear** **High Risk**	**Support for Judgment**
Selection bias		Restrictive inclusion criteria, low number of patients included in study for a 6-month study at a 2000 bed hospital, gender imbalance
Bias in definition and measurement		GCS ≤13
Outcome measurement bias		
Handling of missing data		
Confounding		
Bias of statistics or presentation of result		

**First Author (Year)**	**Williams, J.M. (2017) [59]**
Title	SIRS, qSOFA and organ dysfunction insights from a prospective database of emergency department patients with infection
Journal	Chest
Reviewer	RL, KH, LL,
Study sponsor	Queensland Emergency Medicine Research Foundation
Study type	Prospectively collected data retrospective cohort (October 07–May 11)
Location	Australia
Participants Number Male/Female Age (Median) Patient group	8871 analyzed 51.3% male 49 (30–69) ED patients with suspected infection
qSOFA criteria	Respiratory rate ≥22 bpm, systolic blood pressure ≤100 mmHg, and Glasgow Coma Score (GCS) ≤13
Primary outcome Other outcomes	30-day mortality 1-year mortality
Results	30-day mortality AUC 0.78 (95% CI 0.76–0.81)
Note	

**Risk of Bias**	**Author’s Judgment** **Low Risk** **Unclear** **High Risk**	**Support for Judgment**
Selection bias		
Bias in definition and measurement		GCS ≤13
Outcome measurement bias		
Handling of missing data		
Confounding		Not stated
Bias of statistics or presentation of result		Primary outcome ROC presented in online supplementary material

**First Author (Year)**	**Hu X et al. (2017) [35]**
Title	A multicenter confirmatory study about the precision and practicability of Sepsis-3. [Chinese]
Journal	Chin Crit Care Med (Zhonghua Wei Zhong Bing Ji Jiu Yi Xue)
Reviewer	RL, KH, LL
Study sponsor	National Natural Science Foundation for Young Scientists of China
Study type	Retrospective January 15–June 15
Location	Zhejiang, China
Participants Number Male/Female Age (Median) Patient group	1420 recruited, 329 analyzed 62.6% ? qSOFA-positive ICU patients
qSOFA criteria	Not specified
Primary outcome Other outcomes	28-day mortality
Results	AUC 0.597 (95%CI 0.524–0.669)
Note	

**Risk of Bias**	**Author’s Judgment** **Low Risk** **Unclear** **High Risk**	**Support for Judgment**
Selection bias		Sepsis-3 criteria was used to recruit; high variability from hospital to hospital; ICU patients only
Bias in definition and measurement		qSOFA was not defined, particularly for altered mentation; unclear time point of qSOFA
Outcome measurement bias		
Handling of missing data		
Confounding		Retrospective, high male %, patient characteristics not included
Bias of statistics or presentation of result		Poor and selective presentation of data

**First Author (Year)**	**Wang S et al. (2007) [58]**
Title	Predictive value of four different scoring systems for septic patient outcomes: A retrospective analysis with 311 patients. [Chinese]
Journal	Chin Crit Care Med (Zhonghua Wei Zhong Bing Ji Jiu Yi Xue)
Reviewer	RL, KH, LL
Study sponsor	National Natural Science Foundation for Young Scientists of China
Study type	Retrospective July 12–June 16
Location	Chenzhou, China
Participants Number Male/Female Age (Median) Patient group	311 69.5% 63 ± 17.3 SIRS and suspected infection
qSOFA criteria	Not stated
Primary outcome Other outcomes	28-day mortality Mechanical ventilation, LOS ICU
Results	qSOFA AUC 0.604 SN 0.4 SP 0.78
Note	

**Risk of Bias**	**Author’s Judgment** **Low Risk** **Unclear** **High Risk**	**Support for Judgment**
Selection bias		Inclusion criteria: SIRS and suspected infection; only ICU patients
Bias in definition and measurement		Altered mentation defined by GCS, but did not specify at what level
Outcome measurement bias		
Handling of missing data		Patients with missing value excluded, did not report the number of patients excluded
Confounding		Male-to-female ratio of 2:1
Bias of statistics or presentation of result		Logistic regression double counting variables Poor presentation of table margin
